# Neural Elements for Predictive Coding

**DOI:** 10.3389/fpsyg.2016.01792

**Published:** 2016-11-18

**Authors:** Stewart Shipp

**Affiliations:** ^1^Laboratory of Visual Perceptual Mechanisms, Institute of Neuroscience, Chinese Academy of SciencesShanghai, China; ^2^INSERM U1208, Stem Cell and Brain Research InstituteBron, France; ^3^Department of Visual Neuroscience, UCL Institute of OphthalmologyLondon, UK

**Keywords:** generative model, prediction error, precision, canonical microcircuit, hierarchy, forward pathway, feedback inhibition, interneuron function

## Abstract

Predictive coding theories of sensory brain function interpret the hierarchical construction of the cerebral cortex as a Bayesian, generative model capable of predicting the sensory data consistent with any given percept. Predictions are fed backward in the hierarchy and reciprocated by prediction error in the forward direction, acting to modify the representation of the outside world at increasing levels of abstraction, and so to optimize the nature of perception over a series of iterations. This accounts for many ‘illusory’ instances of perception where what is seen (heard, etc.) is unduly influenced by what is expected, based on past experience. This simple conception, the hierarchical exchange of prediction and prediction error, confronts a rich cortical microcircuitry that is yet to be fully documented. This article presents the view that, in the current state of theory and practice, it is profitable to begin a two-way exchange: that predictive coding theory can support an understanding of cortical microcircuit function, and prompt particular aspects of future investigation, whilst existing knowledge of microcircuitry can, in return, influence theoretical development. As an example, a neural inference arising from the earliest formulations of predictive coding is that the source populations of forward and backward pathways should be completely separate, given their functional distinction; this aspect of circuitry – that neurons with extrinsically bifurcating axons do not project in both directions – has only recently been confirmed. Here, the computational architecture prescribed by a generalized (free-energy) formulation of predictive coding is combined with the classic ‘canonical microcircuit’ and the laminar architecture of hierarchical extrinsic connectivity to produce a template schematic, that is further examined in the light of (a) updates in the microcircuitry of primate visual cortex, and (b) rapid technical advances made possible by transgenic neural engineering in the mouse. The exercise highlights a number of recurring themes, amongst them the consideration of interneuron diversity as a spur to theoretical development and the potential for specifying a pyramidal neuron’s function by its individual ‘connectome,’ combining its extrinsic projection (forward, backward or subcortical) with evaluation of its intrinsic network (e.g., unidirectional versus bidirectional connections with other pyramidal neurons).

## 1. Introduction

Predictive coding theories of brain function have diverse roots; knowledge of hierarchical cortical structure, allied to considerations of the nature of perception, Bayesian formulations of probabilistic representation and constructs borrowed from information theory and statistical physics (Helmholtz, 1860/1962; Gregory, 1980; Mumford, 1992; Dayan et al., 1995; Rao and Ballard, 1999; Lee and Mumford, 2003; Knill and Pouget, 2004; Friston, 2010). Whilst the various algorithms implementing these ideas may differ in their computational strategy (Spratling, 2016), they share a common set of tenets: that the brain intrinsically generates a model of the world in which it finds itself (both the external and internal milieu) which is refined – but not driven – by sensory data. That model is a ‘prediction’ in the sense that it is a guess; a best guess or a Bayesian optimal estimate based simultaneously on both sensory data and prior experience. The model is hierarchical, reflecting the serial organization of the cerebral cortex; higher levels are abstract, whereas the lowest level amounts to a prediction of the incoming sensory data. The actual sensory data is compared to the predicted sensory data, and it is the discrepancies, or ‘error’ that ascends up the hierarchy to refine all higher levels of the model. Thus begins an iterative process: the model optimizes as error minimizes.

Curiously enough our academic understanding of the brain may profit from a similar iterative strategy. It cannot be driven by neuroscientific data alone: brain structure is too complex to infer its workings solely by painstaking observation and scrupulous simulation (Markram et al., 2015). The empirical must be complemented by a top–down theory, applying principles of complex systems function. The free-energy formulation of generalized predictive coding (gPC) is one such theory (Friston and Kiebel, 2009), offering much promise as a basis for understanding the operations of the cerebral cortex in health (Bastos et al., 2012; Adams et al., 2013a) and disease (Adams et al., 2013b; Lawson et al., 2014) and hence with natural extensions to subcortical loops, too (Kanai et al., 2015; Schwartenbeck et al., 2015). Yet, until such time as the nature of neural computation and the fundamental nature of the neural code are completely understood, it will be unrealistic to directly register the computational theory with neurophysiology, and that is not the aim here. What is presented is a more panoramic co-consideration of cortical anatomy and gPC algorithm. Which elements of neural circuitry match the thrust of the computational strategy? Equally, can we identify neural circuit elements omitted from computational interpretation?

The terms of reference for this enquiry will be limited to sensory inference, omitting the extension of gPC theory to motor systems (Friston et al., 2011). As proposed previously, the fundamental ‘agranular’ architectural characteristic of motor cortex can be considered a developmental adaptation to the minimization of prediction error through action, with consequent recession of the input layer for the ascending pathway, granular layer 4 (Adams et al., 2013a; Shipp et al., 2013) and modification of intrinsic microcircuitry (Godlove et al., 2014; Beul and Hilgetag, 2015; Ninomiya et al., 2015). As gPC is proposed as a universal theory of cortical function the ideal should be to analyze the workings of a generic vertebrate (mammalian) sensory cortex (Shipp, 2007). In practice this will be limited to a compilation of circuit data from the two orders that are the most intensively studied, rodents and primates, with a focus upon primary and non-primary visual cortices. Admittedly there are differences in cortical development and organization between the two orders (Dehay et al., 2015) that might, in time, lead to a principled comparative analysis of the implementation of gPC. These differences will create a level of unidentified ‘noise’ in a generic treatment that must be tolerated, for the present, as rapid advances in the knowledge of neural circuitry in transgenic mice cannot yet be replicated in primates.

## 2. The Computational Strategy of Predictive Coding

A percept can be regarded as a hypothesis that explains sensory input. As a result we can see more than meets the eye, as in the example of amodal completion, where we perceive a whole occluded object as opposed to a mosaic of fragments (Kanizsa, 1979). This is plainly based on prior experience of the sensory world, leading to expectation or prediction of sensory regularities. Heuristically, we see what we expect to see. Classic 3D illusions provide striking examples of images betraying expectation and leading to anomalies (e.g., the ‘impossible’ Penrose triangle, or a concave face-mask appearing to be convex and following the movements of the observer). The debilitating effects of a lack of expectation are apparent in trying to make out the words of an unfamiliar language, or even to catch an unfamiliar name.

Illusions and bistable images bring out another aspect of perception, the multiplicity of possible interpretations. Analytically, of course, a 3D configuration is never uniquely specified by its 2D (retinal) image, so some degree of guesswork is obligatory. Conversely, however, it is possible to specify the 2D image created by an imagined 3D object and this is precisely what predictive coding requires of the brain: a model of how objects generate sensory data, referred to as a ‘generative model.’ This modeling capacity must generalize across situations where the object is viewed from any aspect, at any distance, and under any conditions of illumination. Furthermore, it has a temporal element too, extending to predictable trajectories, rotations or regular patterns of ‘biological’ motion – modeling a ‘scenario,’ rather than a snapshot of a visual scene.

The generative model rests on empirical Bayes and is necessarily hierarchical (Friston, 2005). Supposing a hierarchical system such as the visual system to embody a generative model, the percept corresponding to a particular object is not specified at only one level, but has multiple levels of representation. In the hierarchy of face processing, for example, a top-level face area may encode view-invariant face identity, whereas lower levels are more view specific but less identity specific (Freiwald and Tsao, 2010). In addition, because face cells are size and position invariant (Rolls, 1992), it falls to lower areas to represent the spatiotopic ‘filled-in surface’ and ‘border ownership’ attributes of a percept (Qiu et al., 2007; Pollen, 2008; Poort et al., 2012). In short, the gestalt of a ‘face’ comprises a hierarchically distributed representation in which the top-level face area provides the highest stamp of recognition, providing the context for inference of physical attributes in lower-level areas.

In order to recognize an object such as a face from the sensory qualities of its image on the retina it is necessary to ‘invert’ the generative model. This brings us to the core of the computational strategy of predictive coding – the serial, reciprocal exchange of predictions and prediction errors. Signals descending the hierarchy via backward connections between cortical areas are attributed with conveying predictions; signals ascending the hierarchy carry forward the error in those predictions (Mumford, 1992; Rao and Ballard, 1999; Friston, 2008). The representation at any given level attempts to predict the representation at the level below; at the lowest level this amounts to a prediction of the raw sensory input. It is the backward connections, therefore, that instantiate the generative model. Inversion of the generative model describes the process whereby each level generates a prediction-error from the comparison of the state of the representation at that level with the incoming (descending) prediction, and sends this error signal forward, in order to modify the representation in the level above. This process, performed iteratively, results in the minimization of prediction-error at every level of the hierarchy and the consequent refinement of the pan-hierarchical object representation, enhancing the accuracy of recognition.

## 3. The Computational Units Of gPC

The free-energy formulation of predictive coding can be regarded as a generalization of the scheme advanced by Rao and Ballard (1999), estimating both the mean and variance of states of the external world. The ‘free-energy’ itself, that the brain is pictured to minimize, is a measure derived from information theory; in the context of a brain’s generative model interacting with the external world, free-energy is equivalent to a weighted summation of squared prediction-errors (Friston, 2008, 2009). Discussion of the computational strategy of gPC revolves around three quantities that the model is seeking to optimize. These are expected ‘causes,’ ‘states,’ and ‘precisions.’

The reference to ‘expectation’ simply denotes the fact that the model encodes a probabilistic representation of the world, optimized in a Bayesian fashion. In this representation ‘causes’ are invariant aspects of the environment that create regularities in sensory data, such as objects in the visual scene (henceforth referred to as ‘scenic causes’). Their correspondence to elements of the scene is relatively definite at lower levels of the hierarchy (e.g., a contour), and increasingly abstract at higher levels (e.g., a smile). Whereas causes model categorical aspects of the world, ‘states’ model their dynamics; that is, momentary changes caused by the interactions among causes (e.g., motion of an object) or between cause and context (e.g., a rotating object and its illumination). Finally, ‘precision’ measures the reliability of causes and states (specifically, precision is the inverse variance of random fluctuations in these quantities).

**FIGURE 1:**
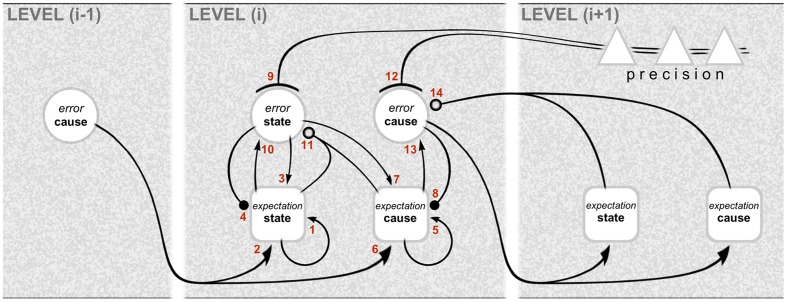
**Graphical representation of the computational interactions between expectation and error units.** The interactions depicted here are based on the differential equations describing the neuronal dynamics implied by generalized predictive coding (e.g., Equation A3 in Kanai et al., 2015). Note the hierarchical structure: predictive coding involves recursive interactions among an arbitrary number of hierarchical levels, of which just one, level (i), is shown in full here. There are separate expectation and error units for causes and states (for definitions, see main text). The computations relating to causes and states are similar, except that the updates for causes are based on reciprocal exchanges between levels. In this scheme, expectation units recursively update their activity (1 and 5) with input from error units associated with other expectations (2, 3, 6, and 7), and predictions about themselves (4 and 8). The error units compare the activity of their associated expectation (10 and 13) with predictions based on a non-linear function of other expectations (11 and 14); note that, for causes, this is a comparison of the expectation arising from the same level (13) with a prediction descending from the higher level (14). Crucially, the gain of error units is modulated by precision signals (9 and 12), shown here to originate from the higher level. The relation with neural architecture is given in **Figure 3**. As portrayed here, the different computational units represent multilaminar neuronal ensembles: expectation units are square, error units are circular, and units mediating precision are triangular. Connections with closed arrowheads are excitatory; connections with closed balls are inhibitory and linear; connections with open balls are inhibitory and non-linear; and connections with arcs have a modulatory (gain) effect. Figure reproduced from Shipp et al. (2013).

The computational architecture of gPC is summarized in **Figure 1**, showing the interactions across three successive hierarchical levels between five kinds of computational unit: these are expectation and error units for causes and states, and units signaling expected precision. Expectation units encode the causes and states describing objects and events (scenarios) in the environment, whereas error units report inconsistencies between expectations. Note that it is error units for causes that are particularly associated with interactions across levels: the emission of forward-going error signals, and the reception of backward going expectation (prediction) signals. By contrast, the exchanges between expectation and error units for states are intrinsic to any given level. The mathematical niceties of the computational interactions between these units are crudely denoted by different types of connector endings (arrowheads, etc.) – sufficient for a treatment of computational architecture as opposed to computational physiology. Error units, for instance, receive a mix of excitatory and inhibitory inputs from expectation units: this is what is necessary, in principle, to affect a comparison and compute the error, but the nature of the synapatology is not precisely specified. Both cause and state error units are shown to receive descending precision signals that are regarded as modulatory: the units encoding expected precision modulate the gain of error units and endow their signaling with greater or lesser weight. This cortical gain control balances the influence of prediction errors at different levels in the hierarchy. Accordingly, precision is associated with the top–down deployment of attention in the sensory domain and with action selection in the motor domain (Friston et al., 2011; Adams et al., 2013a).

Already, the arrangement of computational units in **Figure 1** hints (deliberately) at a certain correspondence with cortical anatomy. The challenge ahead is to make this correspondence explicit, and nominate particular classes of cells and circuits to implement these computations.

## 4. Predictive Coding, Hierarchy and the Canonical Microcircuit

From the outset, predictive coding schemes have equated error units with the superficial (‘supragranular’) source of forward connections, and prediction units (here termed expectation units) with the deep (‘infragranular’) source of backward connections (Mumford, 1992; Rao and Ballard, 1999; Friston, 2005, 2008). All these neurons, the sources of *extrinsic* corticocortical connections (made by axons passing through white matter) are, of course, excitatory pyramidal neurons. To lend historical context to this assignment, it is the different laminar origins of forward and backward connections, coupled to their differential laminar terminations, that provide us with an objective definition of forward/backward (Rockland and Pandya, 1979) – as opposed to more tendentious concepts of ‘forward’ as leading away from primary sensory cortex, or toward cortical areas operating more sophisticated forms of processing. This anatomical principle allowed construction of a multi-tiered hierarchy of cortical areas (Maunsell and Van Essen, 1983; Felleman and Van Essen, 1991), whose anatomical characteristics have subsequently been refined and quantified (Barone et al., 2000; Markov et al., 2014).

**FIGURE 2:**
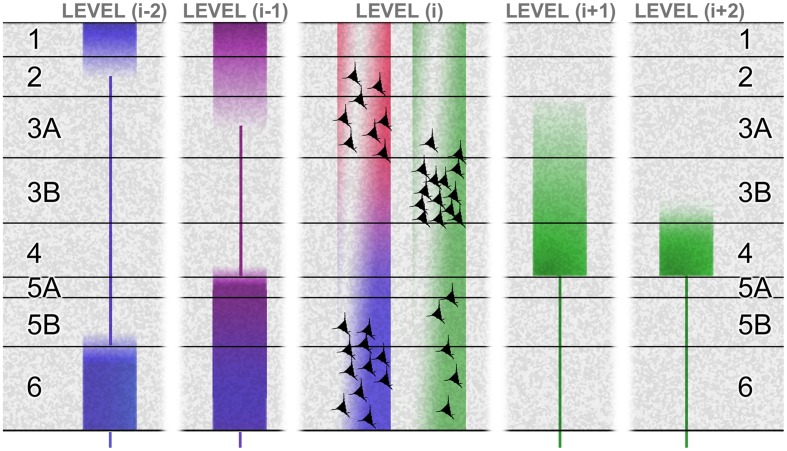
**The laminar basis of hierarchy.** This schematic shows the laminar sources and distributions of ascending connections (green) and descending connections (red, violet, and blue), originating from a certain level (i) in a hierarchical chain. The basic laminar patterns distinguishing ascending and descending connections were originally established by studies of primate visual cortex (Rockland and Pandya, 1979). Systematic variations with hierarchical distance were later formulated as a ‘distance rule’ (Barone et al., 2000; Markov et al., 2014): with regard to origins, the proportion of superficial (layer 2/3A) neurons forming a backward projection decreases with greater distance spanned by the projection (Perkel et al., 1986; Sousa et al., 1991; Rockland and Van Hoesen, 1994; Barone et al., 2000) illustrated by the ‘red’ terminals failing to contact level (i-2). The backward projection originating from deep layers reaches further, but these descending terminations (blue) show a progressive shift of focus upon layers 1 and 6 (Rockland et al., 1994). In the opposite direction, levels (i+1) and (i+2) show a progressive shift of focus of ascending terminations (green) upon layer 4 (Rockland and Pandya, 1979). The differential contribution of superficial and deep sources to superficial and deep terminations in descending projections is not well established. At minimum, the rule may be that like connects with like, laminar-wise. Layer 6, for instance, receives its densest input from layer 6 of the higher area (Henry et al., 1991; Coogan and Burkhalter, 1993). However, layer 1 can receive descending input from deep layers in systems as diverse as primate visual and rodent somatomotor cortex (Cauller et al., 1998; Angelucci et al., 2002), and layer 5 can receive descending input from superficial sources, at least in cat and rat area V1 (Henry et al., 1991; Coogan and Burkhalter, 1993). These patterns are summarized by the violet tone of descending terminations to layer 5 and superficial layers in level (i-1), indicating a mix of superficial (red) and deep (blue) sources from level (i). The bluing of terminals in deeper layers of level (i-1), and all layers in level (i-2), indicates a progressive domination of deep layer sources from level (i). Figure reproduced from Shipp et al. (2013).

**Figure 2** summarizes these hierarchical characteristics as they are known for the primate brain, including the tendency for the laminar origins and terminations of forward and backward pathways to become more distinct for connections spanning more than one hierarchical tier. It also shows (especially for connections between adjacent tiers) that the laminar origins of forward and backward connections are *not* fully compartmentalized to reside above and below the granular layer (layer 4). The superficial layers give rise to both forward and backward connections, though the cells of origin are largely segregated into layers 3B and 2/3A respectively. And, a component of the forward projection arises in the deep layers. These forward-projecting deep-layer cells intermingle with the larger backward-projecting component. Though these mixed projection targets of the superficial and deep layers have been recognized for some time, the fact that forward and backward projections are separate at the cellular level has only recently been demonstrated directly (Berezovskii et al., 2011; Markov et al., 2014). This is significant, as the emission of distinct forward and backward signals is in accord with the tenets of predictive coding theory, whereas a notional broadcast of the same signal – via individual cells projecting in both directions, through axon bifurcation – is not.

Cerebral hierarchy in the rodent brain merits a separate description, because it is different. Rats and mice are alike in that the separate populations of forward and backward corticocortical neurons do *not* have distinctive laminar distributions; both are concentrated in the superficial layers, with diminishing density through all deeper layers – including layer 4, unlike primates (Johnson and Burkhalter, 1994; Berezovskii et al., 2011). Hierarchy is therefore diagnosed by an inverse laminar distribution of the termination of connections, with the backward projection avoiding layer 4 (as with the primate) and the forward projection centering on layer 4 but less focally than primate, being equally dense in layers 2–5, with some spread into layers 1 and 6 (Coogan and Burkhalter, 1993). Furthermore the supragranular layers are comparatively thinner in rodents (Hutsler et al., 2005; Dehay et al., 2015). These details matter when considering rodent microcircuity (that has gained impetus from murine transgenics). Laminar differences in the organization of hierarchy in rodents may signal some departure from the canonical intrinsic circuitry typical of carnivores and primates.

What has become known as the ‘canonical microcircuit’ deals with the onward *intrinsic* relay of the forward pathway, once it has terminated in layer 4 (Gilbert, 1983; Douglas and Martin, 1991; Thomson and Bannister, 2003); details of the circuitry were largely established in primary visual cortex for the onward relay of geniculate signals, but the same basic microcircuit is also observed in non-primary sensory cortex (Lund et al., 1981; Yoshioka et al., 1992; Fujita and Fujita, 1996; Shipp, 2007). In brief, the canonical circuit is an excitatory relay from layer 4 to layer 2/3, and thence from layer 2/3 to layer 5; a relay from layer 5 to layer 6 is typically shown as the final link in the chain, but this projection is spatially diffuse and lighter, lacking the focused ‘columnar’ nature of the preceding two relays (Thomson, 2010). A secondary principle is the lack (or comparative rarity) of reverse excitatory feedback (i.e., pyramid to pyramid) at any of these steps (Thomson and Lamy, 2007). Consideration of the canonical circuit led to the proposal that expectation units would first be generated in layer 2/3, upon receipt of the ascending error signal relayed from layer 4, and that these units should lack an extrinsic projection (Bastos et al., 2012). The subsequent relay to layer 5 would then explain the presence of expectation units in the deep layers, whence they emit the backward prediction signal (Bastos et al., 2012).

There is no equivalent ‘canonical’ circuitry to describe the intrinsic processing of afferents received from the backward connection. This connection is more bivalent: it has both bipolar sources (superficial and deep layers) and bipolar terminations. Furthermore, there remains some uncertainty as to the relative distribution of each source to each termination zone (see **Figure 2**). However, it has been proposed that the superficial component of the backward projection may perform the transmission of precision signals, whereas the deep component may convey descending predictions (Shipp et al., 2013). The grounds for this provisional assignment will emerge as we work through the various stages of cortical processing in greater detail.

**FIGURE 3:**
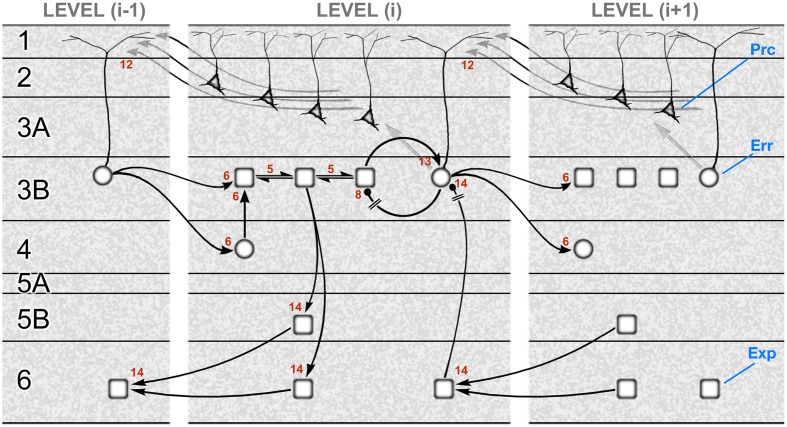
**A template circuit diagram to implement predictive coding.** A neural interpretation of the computational architecture shown in **Figure 1**. The schematic is modeled upon a non-primary area of primate visual cortex, such as area V2 or V4. It shows the correspondence of a subset of the computational interactions in **Figure 1** – those updating scenic causes – to circuit elements bearing the same numbers. These neural pathways include both extrinsic and intrinsic connections, some of them polysynaptic. The extrinsic connections are hierarchical and involve reciprocal connections of one area, level (i), with the levels above (i+1) and below (i–1). The intrinsic connections form a canonical microcircuit that has been summarized as successive relays from layer 4 to 3 (and 2), and 3 to 5, with layer 6 receiving more diffuse input from the layers above. All computational units are designated to be subclasses of excitatory pyramidal neurons: expectation units (rectangular, ‘Exp’) prediction error units (circular, ‘Err’), or precision units (triangular, ‘Prc’). Two pathways (8 and 14) are explicitly indicated to require inhibitory transmission, but all pathways are likely to subsume contacts on both pyramidal neurons and interneurons. The circuitry may be summarized as follows. Forward, extrinsic connections terminate in layers 4 and 3B (pathway 6). Activity in expectation units is maintained by recurrent interactions between these units (pathway 5) and is informed by prediction error signals: excitation by the ascending prediction error (pathway 6) and inhibition by local error units in layer 3B (pathway 8), which are also the source of ascending prediction error routed to the level above (pathway 6). These superficial error units in layer 3B implement a subtraction of two signals: an excitatory signal received from their associated expectation units (pathway 13) and a descending prediction, relayed by a local interneuron (pathway 14). Interlaminar connections from layer 3 to layers 5 and 6 may possibly transmit all three classes of signal, but only the relay of expectation signals is shown here. These contact the deep pyramidal neurons that are the source of descending predictions (pathway 14). Precision signals are shown to arise from layers 2 and 3A, and form a descending chain of transmission through the superficial layers (pathway 12); these signals are capable of modulating pyramidal error units via their apical dendrite. Figure reproduced with modification from Shipp et al. (2013).

**Figure 3** shows a schematic translating the gPC algorithm of **Figure 1** into the neural architecture specified by hierarchy and the canonical microcircuit, as formulated for a mid-tier area of sensory cortex (e.g., primate V2 or V4). Or, to be specific, the subset of computations pertaining to causes, not states; the latter are excluded for the sake of simplicity. The reduced scheme is at once minimalist, omitting a large amount of documented microcircuitry, and exploratory, in that several features of its construction cannot yet be verified. It is therefore intended as a provisional template, constructed from familiar circuit motifs as a plausible model of cortical function. The rapid, recent progress in elucidating details of cortical microcircuitry exploits the new-found ability to record and manipulate specific classes of interneuron, and laminar-specific subclasses of pyramidal neurons in transgenic mice. The majority of this work continues a previous tradition of focusing upon primary visual and sensorimotor areas (V1, S1 and M1). The primary sensory areas have greater laminar complexity, and a particular organization of corticithalamic loops due to their interaction with a first-order thalamic nucleus (Sherman and Guillery, 2011). All this obliges us to tread rather carefully in evaluating the evidence in support of a generic scheme as it might operate in primary and non-primary cortex alike – and in a secondary objective, to begin to consider how known circuit elements lying outside the immediate framework of the scheme might relate to its functional logic.

We will review the evidence underlying the gPC template shown in **Figure 3** in six sections: (1) the identification of separate stages in the forward pathway through the superficial layers; (2) recurrent processing and (3) negative feedback within the forward pathway; (4) the intrinsic relay to the deep layers, and the generation of the backward pathway; (5) the reception and processing of backward predictive signals, and (6) the generation and reception of backward precision signals.

## 5. The Forward Pathway Through Layer 4 and Layer 3

This segment of the model addresses the ascending relay of error signals, and their serial assimilation by expectation and then error units, prior to onward transmission of a higher error signal (pathways 5, 6, and 13 relating to the computation of causes in **Figures 1** and **3**). The architecture of primate area V2 illustrates several of the basic elements, including: (a) projections from V1 to V2 that focus upon layer 4, with individual axons terminating exclusively in layer 4, or layer 3, or arborising across both layers (Rockland and Pandya, 1979; Rockland and Virga, 1990); (b) the description (from Golgi-stained material) of pyramidal granule cells in layer 4 of V2 with axons rising to superficial layers, and of ‘local’ pyramidal cells in layers 3 and 2 with collateral axons contributing to a horizontal fiber plexus in layer 3B, but which lack an extrinsic axon descending below layer 3 (Valverde, 1978; Lund et al., 1981); (c) the concentration within layer 3B of pyramidal cells emitting forward projections from V2 to areas such as V4 and V5/MT (Shipp and Zeki, 1989b; Zeki and Shipp, 1989). One of the major purposes of the present exercise is to identify neural characteristics for matching expectation and error units to separate populations of pyramidal cells; hence the absence, or not, of an extrinsic (forward-projecting) axon is an important diagnostic feature, since superficial expectation units are conjectured to make only local connections, and error units must project forwards. In V2, however, we lack detailed knowledge of recurrent circuits between pyramidal cells, obliging the consideration of other systems.

The termination of the ascending pathway, from V1, in both layer 4 and layer 3 of V2 is typical of the generic forward projection; indeed, the layer 3/4 border is quite indistinct in this respect, as the basal dendrites of layer 3 pyramids commonly ramify within the layer 4 neuropil (Lund et al., 1981). In consequence, the gPC template shows both direct and indirect routes from the error units of one area to the expectation units of a higher area (pathway 6). For consideration in another system, we can compare these two routes to the distinct magnocellular and parvocellular relays from the LGN through V1 to areas V5, and V4 respectively [– a comparison that implicates the LGN as a source of error signals, an issue tackled later]. Magnocellular output from the LGN terminates in layer 4Ca of V1 upon spiny stellate cells that, unlike LGN magnocells, are tuned for orientation and/or direction (Blasdel and Fitzpatrick, 1984; Livingstone and Hubel, 1984a; Hawken et al., 1988; Gur et al., 2005). These stellate cells make only intrinsic connections (Yabuta and Callaway, 1998), and must be accounted expectation units representing the spatiotemporal properties of contours defined by luminance contrast. Their axons terminate in layer 4B, amongst other layers, where their targets include large stellate cells and a smaller number of large pyramidal cells that project directly to area V5 (Shipp and Zeki, 1989a; Nassi and Callaway, 2007); these cells, known to possess complex, direction-selective receptive fields (Movshon and Newsome, 1996) are the candidate error units. This amounts to a disynaptic relay through V1; it is analogous to the more direct route for the ascending pathway shown by the gPC template in **Figure 3** except that the expectation units are formed in layer 4 rather than layer 3.

**FIGURE 4:**
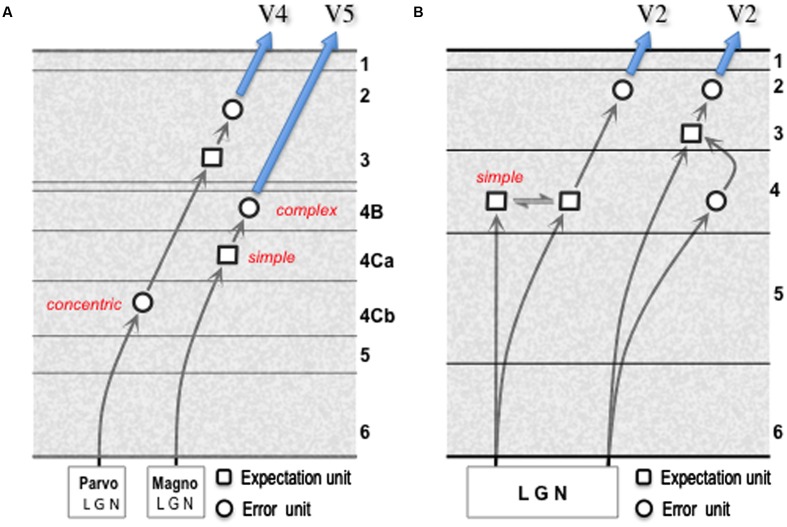
**Models of geniculate transmission through primary visual cortex (V1).** **(A)** Minimal multi-synaptic transmission for signals from parvocellular and magnocellular layers of primate LGN. The two pathways are documented by numerous experimental studies (see main text for details). These enable identification of ‘concentric,’ ‘simple’ and ‘complex’ cells as indicated; the functional character of second and third order cells in the parvocellular pathway has yet to be specified. **(B)** Model for reception and onward transmission of geniculate signals by V1 of mouse cortex. The pattern of spatial convergence of multiple geniculate afferents upon a ‘simple’ cortical cell is the mechanism proposed for the generation of orientation selectivity (Hubel and Wiesel, 1962) either by elongating the receptive field along the axis of the preferred orientation (Li et al., 2013), and/or by the offset between ON and OFF afferents in the orthogonal axis (Lien and Scanziani, 2013). Each form of linear summation of prediction error signals, here from the LGN (refer to Section 9) is germane to the synthesis of an expectation unit, consistent with the prevalence of simple oriented receptive fields in layer 4 of mouse V1 (Niell and Stryker, 2008). Terminal arborisations of single geniculate axons favor contacts with connected excitatory neuron pairs in layers 4 and 3: this is true for presynaptic/postsynaptic pairs in L4/L4 and L4/L3, but not L3/L3; L3/L4 connected pairs are absent (Morgenstern et al., 2016). The modified gPC template shown here interprets the L4/L4 pair receiving joint input as communicating expectation units, and the L4/L3 pair as an input error unit connecting to an expectation unit. The minimal, multi-synaptic pathway through mouse V1 remains undetermined. The schematic shows onward transmission to ‘V2’ (e.g., area LM in mouse) by error units in layer 2/3, consistent with a disynaptic pathway.

A comparison of the parvocellular relay through V1–V4, with the magnocellular relay to V5, is instructive in several respects (**Figure 4A**). The former incorporates an additional synapse, as demonstrated by transneuronal retrograde labeling by means of rabies virus: following viral injection in V5, cell labeling in layer 4Ca is second-order (disynaptic) as expected whereas following viral injection in V4 labeling in layer 4Cb is third-order (trisynaptic) (Nassi and Callaway, 2006; Ninomiya et al., 2011). Thus, firstly, the parvocellular LGN output terminates upon spiny stellate cells in layer 4Cb that are of similar morphology to those of 4Ca, but whose receptive fields closely resemble LGN parvocellular neurons in their concentric organization and chromatic opponent properties (Livingstone and Hubel, 1984a). The onward, intrinsic transmission from layer 4Cb can be interpreted as an interpolated, higher resolution relay of the LGN error signal. Secondly, the cells contacted by the onward relay from layer 4Cb (as identified by *in vitro* laser photostimulation^1^, intracellular (‘patch-clamp’) recording and subsequent recovery of cell morphology by injection of biocytin) are ‘local’ pyramidal cells in layer 3B, analogous to those described in V2, that lack an extrinsic axon projection (Sawatari and Callaway, 2000). The receptive fields of these cells cannot be studied *in vitro*, but cells in this layer are known to advance spatiotemporal and chromatic tuning beyond LGN cells (Shapley and Hawken, 2011). Thirdly, the same study identified a morphologically distinct set of layer 3B cells as output cells, by virtue of possessing an extrinsic axon; these cells were noted to receive a higher proportion of their excitatory input from the superficial layers of V1 and none of them received a detectable, direct input from layer 4Cb (Sawatari and Callaway, 2000). These second and third order cells in layer 3B are thus candidates for expectation and error units respectively and, overall, the parvocellular pathway through V1 is analogous to the less direct, trisynaptic route for the ascending pathway shown by the gPC template.

Geniculate transmission through area V1 of the mouse suggests a similar ‘mixed’ model for layer 4, but without the sub-laminar segregation. As with primate layer 4Ca, the creation of orientation selectivity in layer 4 from non-oriented input implies the presence of expectation units (Li et al., 2013; Lien and Scanziani, 2013). However, a fraction of orientation tuning in mouse V1 is inherited from its geniculate input (Kondo and Ohki, 2016; Sun et al., 2016), since mouse LGN itself demonstrates intrinsic orientation (and direction) selective responses (Piscopo et al., 2013; Tang et al., 2016). Hence mouse V1 layer 4 could contain (oriented) input error units in addition to expectation units. Both are credible sources of the excitatory output from layer 4 to layer 3. The modified gPC template (**Figure 4B**) requires neither of the onward circuit elements to show excitatory (pyramid to-pyramid) feedback which, as noted above, is negligible in layer 3-to-4 transmission (Thomson and Lamy, 2007; Morgenstern et al., 2016) – an important criterion for recognizing layer 4 as a laminar functional subunit (Shipp, 2007). Now, given the density of geniculate input to layer 4 and lower layer 3 (Kageyama et al., 1990; Morgenstern et al., 2016) and its consequent overlap with forward projecting output neurons (Johnson and Burkhalter, 1994; Petrof et al., 2012), the anatomy of rodent V1 raises the theoretical possibility of a minimal monosynaptic transmission in the forward pathway. This has yet to be demonstrated; the formulations of the gPC template in **Figure 4B** predict that the minimal forward transmission in the mouse is also disynaptic, or trisynaptic.

## 6. Recurrent Processing within the Forward Pathway

The serial connections required for minimal forward transmission are complemented by additional circuit elements, shown by the gPC template as recurrent excitatory connections amongst expectation units and a negative feedback loop between expectation and error units. There is no lack of evidence for each of these basic motifs, but the more taxing question is whether they can be preferentially allocated to separate subpopulations of pyramidal cells that could reasonably equate to expectation and error units.

Recurrent reciprocal connections are shown amongst expectation units (pathway 5, **Figure 3**) because each iteration of the computation of expectation combines the current expectation with prediction error arising from the previous iteration. Thus, each expectation unit in a recurrent network represents a similar scenic cause (as defined above): the reciprocal signaling conveys the current expectation, and prediction error is contributed either by input from layer 4 or by direct contact with ascending afferents from a lower area^2^.

Again, much of the empirical evidence that we can draw upon to examine this scheme derives from primary sensory cortex. There are various indications of patchy, ‘like-to-like’ connectivity in the superficial layers of primate V1. For example connections from blob to blob, or interblob to interblob, in terms of compartments defined by cytochrome oxidase (Livingstone and Hubel, 1984b), or between domains of similar orientation tuning. In the latter case, tracer injection sites are typically surrounded by a uniform halo of labeled afferent axons, non-specifically contacting all orientation domains within a range of a mm or so, giving way to patchy, orientation-specific connectivity over a longer range (2–3 mm) (Malach et al., 1993; Bosking et al., 1997; Stettler et al., 2002). By contrast, patchy intrinsic connections are absent in rodent V1, that lacks orientation domains (Van Hooser et al., 2006). None of these studies distinguish between intrinsic connectivity of pyramidal neurons and interneurons. To study recurrent networks at a finer, cellular level it has been necessary to carry out dual or multiple intracellular recordings, where cell identity is recovered later (by cellular injection with a marker such as biocytin). These recordings examine local networks at the sub mm level, as they are conducted in brain slices, *in vitro*. At first, in the absence of any receptive field information, it was possible to establish only basic details, such as the frequency of simultaneously recorded pairs of pyramidal neurons that were found to be connected (10–25% in layer 2/3) (Thomson et al., 2002). Combining this method with laser photostimulation of remote slice loci, in rat V1, it was possible to show that pairs of connected pyramidal cells in layer 2/3 had a greater probability of receiving joint input from any given stimulation site in layer 4, and/or from nearby sites in layer 2/3, compared to unconnected pairs (Yoshimura et al., 2005). In a similar vein (and as discussed in **Figure 4B**) geniculate afferents are also more likely to co-terminate on connected pairs of pyramidal neurons in layers 4 and 3 (Morgenstern et al., 2016). Together these studies imply the existence of separate, mutually exclusive translaminar networks, although they are blind toward the featural specificity of any such network.

To solve this problem recent studies have applied the technique of dual intracellular (or ‘whole cell’) recording to slices cut from a part of mouse V1 where the visual characteristics of neurons had been obtained previously by calcium imaging of cellular activity *in vivo* (Ko et al., 2011). The art of this method is to target electrodes toward specific, identified pyramidal cells within the imaged volume (a 285 × 285 μm block of layer 2/3). Rodent V1, lacking the orientation domains typical of carnivores and primates (Ohki et al., 2005; Van Hooser et al., 2005), has a local organization of preferred orientation and spatial phase that appears to be random (Bonin et al., 2011). Nonetheless, pairs of pyramidal cells with similar orientation tuning are more likely to be connected – twice as likely, compared to pairs with orthogonal preferred orientations (Ko et al., 2011). This study used not only drifting gratings but also movies of natural scenes as visual stimuli, and found that the correlation in neural response to naturalistic stimuli is a still better predictor – e.g., a threefold difference in the occurrence of connected pairs when the recorded population was halved into the most, and least correlated pairs (Ko et al., 2011). A subsequent study employed reverse correlation of the response to natural stimuli to recover the ON/OFF substructure of pyramidal receptive fields (that determines a cell’s tuning to orientation, phase and spatial frequency) and found that correlation of receptive field structure between a pair of cells provided a third predictor of connection probability, and also of connection strength as indexed by the size of recorded EPSPs (Cossell et al., 2015). Numerically, the strong connections between cells of similar receptive field structure were greatly outnumbered by the weaker connections between cells of dissimilar receptive field structure. However, the former appear to govern the receptive field tuning (e.g., to stimulus orientation), providing the dominant influence upon phase-dependent modulation of membrane potential, according to a computational simulation of the network response to drifting, luminance contrast gratings (Cossell et al., 2015).

The evidence is therefore building that pyramidal cells representing similar elements (or causes) within a scene are linked into sparse recurrent networks which, in rodent V1, are freely interspersed with one another (Lee et al., 2016). Can we determine whether such a network consists of expectation and error units? The gPC template (**Figure 3**) specifies reciprocal connections amongst expectation units, whilst expectation and error units are linked by a unidirectional excitatory connection. Notably, in the experiments outlined above, pairs of pyramidal neurons with bi-directional excitatory connections showed greater coordination of activity than unidirectionally connected pairs. These bidirectional pairs exchanged larger EPSPs with one another, had greater similarity of RF structure and, most significantly, a higher level of correlation in the response to natural stimuli (Ko et al., 2011; Cossell et al., 2015). Whilst networked expectation and error units should, theoretically, show similar selectivity toward features such as orientation, their dynamics should certainly differ, the error response waning as the expectation (i.e., the representation of scenic causes) optimizes. The reported differences between unidirectionally or bidirectionally connected pairs of pyramidal cells are therefore potentially consistent with the gPC template. Classes of pyramidal cell whose axons make systematically fewer contacts with other pyramidal cells are known (West et al., 2006); however, it is important to note that the studies considered above (Ko et al., 2011; Cossell et al., 2015) do not definitively establish a pyramidal cell receiving unidirectional input as belonging to a reified ‘unidirectional’ class, simply because bidirectional connections might have existed with another, unrecorded pyramidal neuron.

Looking ahead, methods for studying these issues are well within the purview of today’s experimental toolkit. For instance, classes of forward projecting (error) units and backward projecting (expectation) units could be marked (or transformed) by retrograde viral vectors, prior to probing their local network connectivity through paired intracellular recordings, or by other means (Velez-Fort et al., 2014; Nassi et al., 2015; Wertz et al., 2015; Ghanem and Conzelmann, 2016). Plainly, a well-lit theoretical backdrop can help to stage, and focus, the experimental plot.

## 7. A Negative Feedback Loop within the Forward Pathway

To recap, the gPC template shows that the expectation encoded at each level in the hierarchy is compared to a prediction sourced from expectation units at the level above. This comparison is performed by superficial error units being excited by networked expectation units, and inhibited by the descending prediction. The steps taken by the backward pathway leading to the suppression of error unit activity are discussed below in Section 10; here, we attempt to trace it one step further, in the form of a subsequent inhibitory link from error unit back to expectation unit (**Figure 3**, pathway 8). This establishes a negative feedback loop between the expectation and error units; equally, it creates a means by which expectation can propagate backward across levels by allowing the descending suppression of error unit activity to influence expectation units at the lower level through disinhibition. Now, given that pyramidal cell sub-populations corresponding to error and expectation units are yet to be satisfactorily distinguished, it is not possible to certify the neural elements of this circuit. It is possible, however, to propose a candidate type of interneuron possessing the required characteristics: the Martinotti cell.

Neurochemical and genetic (transcriptomic) criteria identify three cardinal families of interneurons – GABAergic inhibitory cells – defined by the mutually exclusive expression of three markers, namely parvalbumin (PV – a calcium binding protein), somatostatin (SST – a peptide co-transmitter), or 5HT3a, a serotonin receptor; the third group is also commonly identified by expression of the peptide VIP (Fishell and Rudy, 2012; Pfeffer et al., 2013; Tasic et al., 2016). Again, much of the recent evidence pertains to the rodent brain, but similar traits are apparent in all other species that have been examined. Whereas PV and SST cells contact both pyramidal cells and interneurons, the third class solely contacts other interneurons (i.e., PV and SST cells) (Pi et al., 2013), immediately excluding it from consideration for the role described above. The PV class consists of ‘fast spiking’ interneurons and subdivides into so-called ‘basket’ and ‘chandelier’ cells, defined by cell morphology. The SST class has different spiking characteristics (e.g., accommodating, or bursting) and contacts pyramidal cells upon more distal dendrites. The Martinotti cell is a major subclass of SST interneuron occurring in layers 2–6, that is morphologically characterized by bitufted dendritic trees (Wang et al., 2004). As before, it is a comparison of PV and SST classes’ differing roles in visual function, in rodent V1, that helps to place them within the gPC template.

Considering the superficial layers in particular, PV cells are found to provide feedforward inhibition, related to exerting gain control over incoming signals (Ma et al., 2010; Atallah et al., 2012; Wilson et al., 2012). PV cells in layer 2/3 receive excitatory input from layer 4 and make strong reciprocal, contact with local pyramidal neurons (Yoshimura and Callaway, 2005; Xu and Callaway, 2009; Adesnik et al., 2012). By contrast SST cells in layer 2/3 provide lateral inhibition, related to visual functions such as surround suppression (Adesnik et al., 2012; Nienborg et al., 2013). They do not receive excitatory input not from layer 4, but only from local axon collaterals of pyramidal cells in layers 2/3 (Xu and Callaway, 2009; Adesnik et al., 2012), and consequently show longer latency to activation by visual stimuli (Ma et al., 2010). Importantly, SST cells have receptive fields that are significantly more tuned, than PV cells, to orientation and motion direction of grating stimuli (Ma et al., 2010), a requisite property for representing specific scenic causes. They also have spatially overlapping ON and OFF subfields and show little modulation of activity with the phase of a grating stimulus (Ma et al., 2010), both characteristics of a ‘complex’ receptive field, that they must inherit (or synthesize) from their driving pyramidal cell inputs.

More specific details of network connectivity are harder to establish. One *in vitro* study of rat V1 layer 2/3 combined photostimulation with whole-cell recordings from pyramidal/interneuron pairs (Yoshimura and Callaway, 2005). It distinguished PV cells from non fast-spiking interneurons (i.e., the non-PV class included both SST and VIP types) and found that PV cells were very likely to make strong contacts with pyramidal neurons from which they received an input. Such pairs were also more likely to receive joint input from nearby locations in layer 2/3 (and layer 4) compared to unconnected PV/pyramidal pairs (Yoshimura and Callaway, 2005). Thus the PV cells were shown to link into fine-scale local excitatory networks between layer 4 and 2/3 (– mentioned previously in the context of recurrent connections amongst expectation units, see Section 6). The statistics of pairs of non-PV interneurons and pyramidal neurons proved rather different: 68% showed no connection, 16% a one-way inhibitory connection, 12% a one-way excitatory connection, and just 3.6% pairs were reciprocally linked. Further, connected and unconnected pairs showed no difference in the likelihood of receiving joint input from nearby loci in layer 2/3 (Yoshimura and Callaway, 2005). Another study found that activation of a single SST cell (i.e., here, specifically an SST cell) suppressed activity of 16% of nearby pyramidal cells, on average, compared to an equivalent figure of 43% for PV cell activation (Wilson et al., 2012) – from which it may be inferred that SST cells make more selective contacts, in line with their higher stimulus selectivities (Ma et al., 2010). These characteristics of SST cells are all consistent with the gPC template’s negative feedback loop between error and expectation classes of pyramidal cell, although they cannot be held to demonstrate such a motif. Finally, SST and PV cells were also reported to differ in their inhibitory action upon pyramidal cells (as assessed by considering visual, orientation tuning curves *in vivo* whilst applying optogenetic photostimulation of either PV or SST interneurons): PV cells exerted a divisive effect (i.e., a proportionate reduction in visually driven activity) whereas the effect of SST cells was more subtractive (a fixed reduction, subject to floor effects at poorly responsive orientations) (Wilson et al., 2012). Notably, the gPC template portrays the action of inhibition as a simple subtraction; however, extending the level of analysis to such details of physiology requires caution, as the blanket optogenetic activation of SST cells across trials with varying orientation obscures their natural, physiological profile of stimulus tuning.

In summary, the characteristics of SST/Martinotti neurons lend themselves to the two pathways (8 and 14, **Figure 3**) in the gPC template requiring inhibitory transmission; *ipso facto*, the omission of an overt role for PV neurons within the template is a clear deficit. Taking up the theme of the Introduction, consideration of interneuron diversity should afford some potent, data-driven adjustment of the top–down chain of gPC theory, algorithm and neural implementation.

## 8. From Superficial to Deep: The Generation of Predictions

As noted above, the hierarchical representation of causes develops increasingly abstract forms at higher levels. Therefore, the prediction fed back to a lower level cannot be an immediate, direct relay of the expectation unit activity computed in the superficial layers. According to the gPC algorithm, the expectation only becomes a prediction once it has undergone a non-linear transformation, suitable for the lower level area to which it is directed. A single cause may naturally have many predictive consequences. A high-level expectation of viewing a ‘talking-head,’ for instance, would generate predictions relevant to pathways processing facial structures, expressions, lip movements and speech sounds. The backward transformations required are necessarily non-linear, because they must model non-linear interactions between the causes of sensory data (such as occlusion, in this example lips periodically obscuring teeth). Neurally, the gPC template follows the canonical microcircuit and suggests that the generation of backward going predictions of causes takes place through the transmission of the superficial expectation signal through to the deep layers. This interlaminar intrinsic processing should be non-linear (Bastos et al., 2012), and it must involve both layer 5 and layer 6, as both layers contain backward projecting neurons (Markov et al., 2014). For various reasons, they are best considered separately.

Layer 5 is, in effect, the ‘motor’ layer of cortex as it exclusively houses cortico-subcortical (CS) cells projecting to the spinal cord (from sensorimotor cortex) plus a rather broader distribution projecting to structures such as the pons, or superior colliculus (Guillery and Sherman, 2002). CS neurons also commonly project to the thalamus, through axon collaterals. However, the corticocortical (CC) and subcortically projecting pyramidal neurons are almost totally separate populations (Petrof et al., 2012), with distinct cellular characteristics, both physiological and morphological (Groh et al., 2010; Kim et al., 2015). The presence of a robust intrinsic connection from layer 2/3 to layer 5 is a highly conserved feature across hierarchical level, sensory modality and species (Briggs and Callaway, 2005; Thomson and Lamy, 2007; Weiler et al., 2008; Llano and Sherman, 2009; Hooks et al., 2011). As a principle source of translaminar input to layer 5 it must feed both CC and CS neurons (as well as inhibitory interneurons, and local pyramidal cells that fail to make extrinsic connections). There are indications that CC and CS neurons form intermingled local networks and subnets (Song et al., 2005; Perin et al., 2011; Zarrinpar and Callaway, 2016), that may receive selective contacts from separate networks in layer 2/3 (Kampa et al., 2006). This work, attempting to distinguish the sources of input to recognized classes and subclasses of CC and CS cells, is of limited volume. For present purposes, it also has the major drawback that, to date, studies have been restricted to primary sensory areas – that, by definition, lack backward connections to a lower cortical area. The same restriction, a lack of data from non-primary areas, also precludes any discussion of backward *cortical* projections from layer 6. However, for primary areas, layer 6 is the source of the backward connection to first order thalamic nuclei (Sherman and Guillery, 2011). The premise, under gPC, is that this output also conveys descending predictions and is therefore worthy of further scrutiny.

One such system that has been intensively investigated, both anatomically and physiologically, is the reciprocal communication between the lateral geniculate nucleus (LGN) and area V1 in primates. Five specific types of corticogeniculate neuron have been identified, distinguished by variations in sublaminar position and cellular morphology (Briggs et al., 2016). Two of these (termed ‘Iβ’ and ‘IC’) correspond to particular classes of layer 6 pyramidal neurons whose direct (i.e., monosynaptic) input from other layers of V1 has been studied by means of *in vitro* photostimulation (Briggs and Callaway, 2001). Iβ and IC cells were found to receive rather similar patterns of input from other layers. Referring to the generic gPC template (**Figure 3**) we might anticipate input from layer 5 and/or some direct input from the superficial layers. Adapting the scheme to cater for primate area V1 in particular (as set out by Section 5) there should be input from layer 4Ca and layer 3B, interpreted to derive from expectation units developed separately within the magno- and parvocellular channels; layer 4B would be a lesser source if it is dominated by error units, to the relative exclusion of expectation units. This pattern largely matches what is reported: significant inputs to both ‘Iβ’ and ‘IC’ cells from layers 5, 4C and 2/3, but not from layer 4B (Briggs and Callaway, 2001). Hence the observed pattern of translaminar input is provisionally consistent with expectation being relayed from superficial layers to corticogeniculate neurons, although the precise sources and characteristics of these intrinsic signals are yet to be established.

Examining the data in greater detail, layer 4C was found to provide input from *both* subdivisions, 4Ca and 4Cb (Briggs and Callaway, 2001). Sub-layer 4Cb, unlike 4Ca, was interpreted above as a stage refining the incoming LGN error signal, and lacking expectation units. Therefore the signal relayed to corticogeniculate cells by spiny stellate cells of layer 4Cb should retain this classification as an (incoming) error signal. But the inclusion of such a signal amongst the mixture of inputs integrated by corticogeniculate neurons should not be thought surprising, as corticogeniculate neurons themselves are known to receive direct contacts from LGN axons, either through branching of the apical dendrite in layer 4C or through geniculate axon collaterals in layer 6 (Briggs and Usrey, 2007, 2009). Notably, the receptive fields of corticogeniculate neurons are not simply concentric in organization, like those of layer 4Cb or the LGN itself. They are orientation selective, indicative of representing a visuospatial feature such as a contour (Briggs and Usrey, 2009). Thus, in addition to receiving and processing expectation signals relayed from superficial layers, corticogeniculate neurons might be interpreted to perform a parallel computation of expectation from incoming error signals. This additional function is not greatly at variance with the gPC template, and it could be a particular feature of primary cortex and/or corticothalamic (CT) as opposed to corticocortical circuitry. Furthermore, it prompts us to consider how the LGN processes a descending prediction and generates an error signal by combining it with retinal input.

## 9. Signal Processing in the LGN

The LGN, like all other thalamic nuclei, lacks the complex computational architecture of the cortex. Its intrinsic circuitry is limited to the contacts made by local interneurons, as the excitatory relay cells do not make interlaminar recurrent connections amongst themselves. These two basic cell types share excitatory inputs from retina and cortex, plus inhibitory input from the thalamic reticular nucleus and a variety of modulatory influences from brainstem nuclei (Sherman and Guillery, 1996). Viewed from a top–downward perspective, the LGN is the furthest outpost in a backward chain, since there is no projection leading from LGN to retina. It has therefore been viewed as a model system for investigation of feedback processing, by attenuation or enhancement of corticogeniculate output, in several different species, with varying interpretations of the diversity of effects obtained – not all of them consistent (Cudeiro and Sillito, 2006; Briggs and Usrey, 2011). In line with the aims of this review, the focus here will fall upon a single study (of cat LGN) that elucidates the generation of an error signal according to the principles of predictive coding (Wang et al., 2006).

**FIGURE 5:**
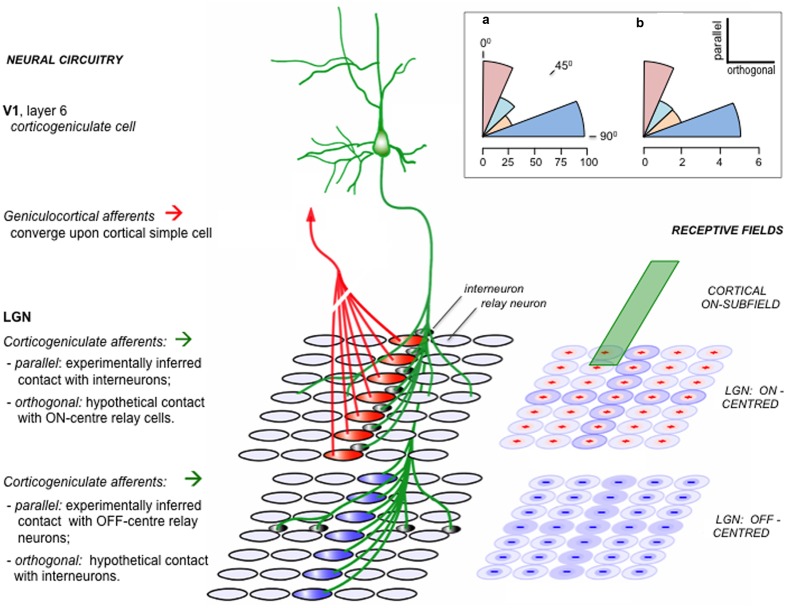
**Functional alignment of corticogeniculate circuitry.** This schematic is based on experimental analysis of corticogeniculate function in the cat [adapted from supplemental data of Wang et al. (2006)]. **Right**: an ‘ON’ subfield of an oriented corticogeniculate neuron (green) is shown to lie in register with a set of LGN receptive fields, schematically subdivided into separate arrays of ON-center and OFF-center units. **Left**: neural circuits amongst these units. Geniculo-cortical convergence from a linear array of LGN relay neurons is thought to be the primary mechanism generating orientation selectivity in cortical neurons. A corticogeniculate unit possessing the ON subfield shown at right, and sharing the orientation specificity determined by the ‘red’ ON-center LGN units, makes indirect contact with these same ‘red’ units via LGN intrinsic interneurons. The same corticogeniculate unit also makes direct excitatory contact with a matching array of ‘blue’ OFF-center units. The reverse-phase specificity of these ‘parallel’ backward connections (i.e., those aligned parallel to the cortical unit’s preferred orientation as transcribed onto the visuotopic map in the LGN) was inferred from pharmacological modulation of burst firing by relay cells during visual stimulation ( - as described in the main text). For each array of LGN units a second set of connections is also shown, in orthogonal alignment. These connections were detected by the same physiological signature as the parallel connections, namely an increment or decrement in burst firing; but, because the receptive fields of the orthogonal LGN relay units did not overlap with the receptive field of the recorded corticogeniculate unit, they were not considered eligible for the in-phase v. reverse-phase classification. Hence the orthogonal connections shown here (direct excitatory to ON-center units, indirect inhibitory to OFF-center units) are conjectural (see ‘Theory’, below). **Inset (a):** the angular distribution of physiologically inferred corticogeniculate contacts; each sector of arc shows the proportion (%) of recorded LGN units with inferred contacts, classified as parallel (0 – 22.5°), intermediate (22.5 – 45°; 45 – 67.5°) or orthogonal 67.5 – 90°.). **Inset** **(b):** the angular distribution of anatomically reconstructed corticogeniculate terminations. Each axon terminal field has an elongated central zone of high bouton density, characterized by the angular relationship to the transcript of its parent neuron’s receptive field upon the visuotopic map of the LGN (Murphy et al., 1999). Each sector of arc shows the number of axon terminal fields so classified. The anatomical pattern shows a bias away from intermediate angles toward either a parallel or orthogonal disposition, similar to the physiological data. *Theory*: One way to interpret the orthogonal component of the corticogeniculate interaction in keeping with the logic of the reverse-phase arrangement and the tenets of predictive coding is to suppose that the orthogonal component enacts another version of suppression by addition to the reverse channel – but now in relation to rival orientation channels. Logically, the orthogonal component should complement the action of the parallel component emitted by the same class of corticogeniculate neuron. Thus, (depicted here as if both the parallel and orthogonal components arise from one-and-the-same neuron) since the cortical ON subfield inhibits parallel ON-center LGN units, it should excite orthogonal ON-center units; equally, it should complement the excitation of parallel OFF-center units by inhibiting orthogonal OFF-center units.

This study succeeded in demonstrating a reverse-phase linkage between the ON and OFF receptive field components of V1 and LGN cells. The nature of experimental inference was quite indirect, and worth examining in more detail. The study recorded simultaneous visual activity in LGN neurons and layer 6 corticogeniculate neurons, whilst periodically administering an antagonist to the GABA B receptor at the cortical site of recording. This enhances the gain of the response to visual stimuli without raising background spontaneous activity. The effect of this pharmacological manipulation of cortex upon LGN activity was monitored by measuring the frequency of LGN burst spiking. Burst firing is a property of all thalamic relay cells, and occurs when relay cells have been hyperpolarized for periods of 100 m or more. Hence a comparison of the ratio of tonic spikes to burst spikes across trials when the drug was applied, or not, provided a sensitive measure of cortical influence upon LGN neurons’ sensitivity to the visual stimuli. The majority of corticogeniculate neurons in cat V1 possess simple receptive fields, characterized by distinct ON and OFF subfields (Grieve and Sillito, 1995). LGN fields are concentric (ON-center/OFF-surround, or the reverse). The precise alignment of the LGN field center with the cortical field was found to determine the polarity of cortical influence (**Figure 5**). Where an LGN ON center coincided with a cortical ON subfield, or where an LGN OFF center coincided with a cortical OFF subfield, the cortical influence was seen to be suppressive (as indexed by an increase in the frequency of burst firing during the period of visual stimulation). Conversely, for receptive field pairs showing ON/OFF, or OFF/ON registration, the cortical influence was facilitatory (indexed by a decrease in burst firing). In some fortuitous cases paired recordings of LGN neurons allowed both effects to be observed concurrently (Wang et al., 2006).

How may we rationalize this finding? The concentric ON/OFF structure of LGN receptive fields can be modeled as a difference of gaussians, and considered to report a local contrast signal – the center of the field is either lighter or darker than its immediate surround. Activity in a corticogeniculate ‘simple’ cell with, say, an ON subfield lying in registration can be considered as a prediction of the relative lightness of that same element of the retinal image; if it acts to inhibit an LGN ON-center cell, this can be considered as generating an error signal, by subtracting the prediction from the incoming sensory data (Jehee and Ballard, 2009). The same logic applies to OFF/OFF registration, in respect of predicting relative darkness. An obvious problem arises in instances where the prediction of relative lightness (or darkness) exceeds the actual image data; LGN neurons, like all neurons, have a zero baseline of activity and cannot code a negative error signal by adopting a negative firing rate. The solution is equally obvious: the corticogeniculate prediction is not only subtracted from the same phase of LGN representation, but added to the opposite phase – exactly as observed for cases of ON/OFF and OFF/ON registration. Thus the outcome can be regarded as a negative error signal by virtue of being transmitted through the ‘opposite’ channel to the prediction, and logically should modify the prediction in reverse manner to a positive error signal.

The functional logic of this reverse-phase arrangement might also extend to the spatial topography of the back connection. Corticogeniculate simple cells are orientation selective, with receptive fields elongated along the axis subdividing their ON and OFF subfields. The axon terminal field has been found to trace the same axis upon the retinal map in the LGN, as if conveying a prediction of contrast along a contour, as opposed to a point in space. This spatial arrangement is illustrated in **Figure 5**, along with an accompanying finding, which is that the corticogeniculate terminal field also contains an orthogonal component (Murphy et al., 1999); it could be said to describe a ‘cross,’ rather than a ‘bar,’ within the LGN map. Remarkably, this anatomical configuration was mirrored by the spatial pattern of the corticogeniculate interaction, with a similar ‘orthogonal’ component being recorded physiologically (Wang et al., 2006). This allows an analogous functional interpretation, now with regard to a reverse action upon rival channels of orientation as opposed to lightness/darkness. See **Figure 5** for further explanation.

## 10. Cortical Processing of Backward Predictions

Theoretical treatments of predictive coding typically stress that the role of backward connections is to suppress prediction error. The immediate problem in translating this idea into neural circuitry is that the source of the back connection is uniformly comprised of excitatory pyramidal neurons. The obvious recourse is to propose that back connections primarily target inhibitory interneurons, but there is no evidence that this is the case. Examined at the ultrastructural level in the rodent visual system, forward and backward going axonal terminals make contact with similar proportions (12–15%) of postsynaptic dendritic structures that can be recognized as belonging to interneurons (Gonchar and Burkhalter, 1999). Forward connections, in fact, are found to exert a stronger excitatory drive than backward connections upon PV interneurons (perhaps to achieve tighter regulation of gain control, as discussed above) (Yang et al., 2013). Another possibility is that the focus of the backward connection upon layer 1 is significant (Bastos et al., 2012), given that the only cells with cell bodies in layer 1 are inhibitory neurons, that receive a slightly higher proportion of the backward terminations than in other layers (20% – the other 80% being formed upon the apical dendrites of pyramidal cells) (Gonchar and Burkhalter, 2003; Anderson and Martin, 2006). Studies investigating various forms of stimulation of layer 1 report a variety of excitatory, inhibitory and disinhibitory effects upon pyramidal cells – the latter reflecting the fact that some layer 1 interneurons inhibit other interneurons (Cauller, 1995; Chu et al., 2003; Shlosberg et al., 2006; Letzkus et al., 2011; Wozny and Williams, 2011; Jiang et al., 2013). These effects can have a center-surround organization (Jiang et al., 2013; Zhang et al., 2014) but there does not appear to be an overall bias of layer 1 circuitry toward pyramidal suppression.

If we accept that the backward pathway exerts a balance of excitation and inhibition upon pyramidal cells, the required suppression of error unit activity is more likely to be achieved by competitive interactions amongst pyramidal cells, mediated by additional, intrinsic interneurons that are not directly contacted by backward terminals. An example is shown in the gPC template, implemented by a connection from the deep layers to superficial error units that will be justified in greater detail below. There is also a more general underlying idea here, that the role of backward prediction can be recast as one of bimodal adjustment of the pattern of error unit activity, as opposed to unimodal suppression. The foregoing discussion of prediction error generated by the LGN illustrates the principle: that a number of cardinal features such as lightness/darkness (in vision – also opponent colors, opposite directions of motion and orthogonal orientations) are encoded antagonistically, and that a backward-going prediction might add to error unit activity in the opposite channel, as well as subtract from activity within its own channel. This ‘crossover’ strategy addresses not just one, but two related difficulties that have been identified in neural schemes for predictive coding (Kogo and Trengove, 2015): the difficulty of achieving suppression via an excitatory backward connection, and the difficulty of coding a ‘negative’ prediction error – instanced when a descending prediction exceeds the magnitude of the expectation expressed at the subordinate level. The latter problem is resolved, in principle, by the addition of this prediction to the error unit activity of a rival channel. The rival error signal, transmitted to the higher level, should enhance the representation of the opposite feature and reduce the original representation by mutual antagonism affected by intrinsic circuits within the higher level. For illustration, suppose there is a descending prediction of leftward object motion within a static scene. It cannot be satisfactorily subtracted from the baseline activity of leftward reporting error units. However, if it were to enhance error unit activity in the opposite channel, reporting rightward motion, the ascending error signal should act to subdue the representation of leftward motion in the higher area causing the prediction.

An additional, circumstantial rationale for this indirect mechanism of adjusting prediction error is that deep layer; backward projecting neurons often connect with not one lower area but several, by means of bifurcating axons (Bullier and Kennedy, 1987; Rockland et al., 1994; Rockland and Drash, 1996; Markov et al., 2014). Two areas might therefore receive the same set of predictive signals, despite representing different causes. As a back-transmitted expectation undergoes a non-linear transformation to become a prediction, each area may be obliged to complete this process internally, before presenting a suitably fashioned prediction to its superficial error units. The polysynaptic pathway indicated by the gPC template (pathway 14) shows a deep layer pyramidal neuron receiving a backward input and communicating with a superficial error unit via an interneuron, so achieving a suppression of prediction error. Amongst the variety of cell types found in the deep layers there are descriptions of ‘local pyramids’ in both layer 5 and layer 6 – pyramidal cells whose axons make only local intrinsic connections, often translaminar and rising to the superficial layers (Wiser and Callaway, 1996; Briggs and Callaway, 2005; Kim et al., 2015). This finding can be paired with one of the ‘rules’ of the canonical microcircuit, the absence of excitatory feedback: a survey of studies with paired intracellular recordings shows that pyramid-to-pyramid connections from layer 5 to layer 3 are generally weak and infrequent (Thomson and Lamy, 2007). Hence the deep pyramidal neurons connecting with the superficial layers may make preferential contact with inhibitory neurons; photostimulation studies have identified specific classes of superficial non-PV interneurons that receive excitatory input from deep layers, particularly layer 5 (Dantzker and Callaway, 2000; Xu and Callaway, 2009).

Alternatively, there can be direct inhibitory input to superficial pyramidal neurons from the deep layers (Dantzker and Callaway, 2000). A subsequent study, again *in vitro*, used focal optogenetic stimulation to examine laminar sources of inhibition in mouse V1, and distinguished a minority of pyramidal cells in layer 2/3 as a class receiving strong input from layer 5 interneurons (Katzel et al., 2011). The gPC template would equate these with error units and the majority class, whose dominant inhibition was intralaminar, with expectation units^3^. The functional role of a similar translaminar pathway in mouse V1 has been investigated by optogenetic stimulation, *in vivo*, of a subset of layer 6 pyramidal neurons (specifically, CT neurons). This is found to drive activity in a population of fast-spiking (PV) interneurons, mainly in layer 6, and subsequently to depress the response to visual stimuli of pyramidal cells in layers 2–5 (Olsen et al., 2012; Bortone et al., 2014). As with other accounts of PV interneuron function, the effect was described in terms of gain control, as it did not totally suppress the visual response, nor alter visual selectivity such as orientation preference or tuning (Olsen et al., 2012). On first inspection this translaminar system well appears to fit the gPC template (bearing in mind that rodents posses a broader laminar distribution of forward-projecting neurons) – but there are also some discrepant aspects. These issues are worth exploring in greater detail, exploiting other evidence obtained in the rodent visual system.

Firstly, on the plus side, there is known to be a ‘deep-to-deep’ component of the backward projection to V1, one that originates in the deep layers and terminates preferentially in layer 6 (Coogan and Burkhalter, 1993). Secondly, the CT cells of layer 6 that were specifically driven by optogenetic stimulation (Bortone et al., 2014) differ in two key respects from the other major group of layer 6 pyramidal cells, corticocortical or ‘CC’ neurons. One is that CT neurons are found to receive a significant proportion of their inputs from higher areas of cortex, whilst CC neurons may project to distant regions of cortex (and not to thalamus, by definition), but receive rather little input from outside their locality of V1 (Velez-Fort et al., 2014). The other is that CT neurons are also found to be highly selective for visual features such as orientation and direction of movement, making them suitable as a vehicle for specific predictions of scenic causes; CC neurons, by comparison, are more broadly tuned for these features (Velez-Fort et al., 2014).

On the minus side, the gPC template indicates that the polysynaptic backward prediction pathway (**Figure 3**, pathway 14) terminates specifically upon error units, but the optogenetically driven depression of visual response appeared to occur, with varying magnitude, in virtually all pyramidal cells in layers 2–5 that were tested (Olsen et al., 2012). Hence the cells recorded should likely have included expectation units as well as error units. It is difficult to predict how expectation units should respond under the experimental condition of blanket optogenetic stimulation, causing simultaneous activation of CT cells that are found to fire selectively and sparsely to visual stimuli under more physiological conditions (Velez-Fort et al., 2014). A second issue, possibly related to the first, is the reported restriction of optogenetically induced second-order spiking activity to deep layer PV neurons (Bortone et al., 2014). As discussed above, the particular characteristics of PV cells render them less suitable for feature-specific interactions with pyramidal cells than other classes of interneuron, such as SST/Martinotti cells. A subsidiary study (*in vitro*) showed that optogenetic stimulation of layer 6 CT cells did in fact cause a general, subthreshold activation of interneurons in layer 2/3 (Bortone et al., 2014). Again, it may be that a blanket network activation propagates more efficiently amongst broadly tuned than narrowly tuned units. If so, the study may have illustrated the principle of translaminar inhibition, but not in a specific sub-system purposed for the adjustment of error unit activity.

Corticothalamic cells in mouse V1 include many (if not all) that project to the LGN, and can be considered expectation units by the same logic that was applied to the corticogeniculate projection in the macaque, discussed above (see Section 8). But there is one final problem, here, that astute readers will already have detected. The gPC template indicates that the expectation units receiving descending predictions, and those emitting them, should constitute separate populations: local pyramids and extrinsic, backward-projecting pyramids. So far, the CT cells receiving descending inputs from higher visual areas, communicating with the superficial layers and (nominally) projecting to the thalamus have been treated as one and the same population. This population is actually the subset of layer 6 neurons expressing cre-recombinase in the *Ntsr1-Cre* transgenic mouse line (and that can thus be selectively transformed to express channelrhodopsin). There is no guarantee that it constitutes a single functional class, and two sub-classifications [on the basis of dendritic arborisation (Olsen et al., 2012) and transcriptomics (Tasic et al., 2016)] have been put forward. Perhaps, like another transgenic line (layer 5 - *Efr3a-Cre*) it includes both local pyramids and projection neurons (Kim et al., 2015).

## 11. The Generation and Action of Precision

The role of ascending prediction error is to modify the representation of scenic causes in the level above, and improve the descending prediction for the next iteration of computation. Yet incoming sensory data, that lies at the root of ascending prediction error, varies in its reliability. Ultimately, the Bayes-optimized representation is determined by the balance of data reliability and the strength of prior expectation. The confidence or, in more physiological terms, the gain associated with prediction error is the gPC quantity known as precision – analogous to the way that the statistical significance of an effect is assessed in relation to its standard error. According to the gPC algorithm, the brain encodes an estimate of precision (‘expected precision’), which is a computation based on the sum of squared prediction error (Kanai et al., 2015). In the gPC template, precision is computed within a stream riding on top of the superficial error units, in the uppermost cellular layers 2/3A. These layers are, of course, the source of the superficial component of the backward projection, so the precision signal computed by an areas can be broadcast backward, in order to control the gain of error signals ascending to that area, as well as error signals computed locally. Precision is also considered to be broadcast and coordinated more globally, because the regulation of precision is seen as equivalent to the psychological construct of selective attention (Feldman and Friston, 2010). So, within the visual system for instance, precision-related signals may be conveyed to the pulvinar via the deep layers, as the pulvinar is well placed to coordinate the gain of ascending pathways through direct effects upon superficial error units, or by regulation of effective connectivity through the induction of coherence in transareal oscillatory relationships (Shipp, 2003; Purushothaman et al., 2012; Saalmann et al., 2012; Kanai et al., 2015). Here, however, the focus is upon the action of precision signals conveyed by the backward cortical pathway.

As noted in **Figure 2**, the superficial component of the backward connection has a short range (in terms of spanning hierarchical tiers) and appears to concentrate its terminals in the upper layers. Layer 1 is the principal target, where the backward afferents may influence neural activity through the profuse apical dendritic arborisations of pyramidal cells. These comprise further populations of backward projecting cells in layer 2/3A (precision units), forward projecting cells in layer 3B (error units) and larger pyramidal cells of the deep layers (potentially also error units). Backward connections to mouse V1 from area LM (equivalent to V2) have been shown activate layer 2/3 pyramidal neurons almost exclusively through the apical, as opposed to the basal dendritic compartment (Yang et al., 2013). Postsynaptic structures in the backward pathway have a rich complement of the NMDA type glutamate receptor that, due to its voltage sensitivity, can have a regenerative effect (‘plateau potentials’) upon cell membrane depolarization. Beyond that, an additional amplifying mechanism has been identified for apical dendrites, conditional upon the presence of a second spike-initiation zone situated near the apical tuft, driven by calcium ion channels (Larkum, 2013). Activation of these dendritic ‘calcium spikes’ dramatically enhances the initiation of axonal spiking, and is itself controlled by an interaction between the apical and basal dendritic compartments. Thus forward pathway input to the basal dendrites may initially trigger some axonal spiking, and consequent back-propagation of the action potential toward the apical tuft, where concurrent activation through backward pathway input may trigger apical calcium spiking. In such a state, the cell is far more sensitive to continued forward pathway input (Larkum, 2013). Whilst this mechanism has specifically been described in large layer 5 pyramidal cells, the assumption is that superficial pyramidal cells may share similar properties, enabling gain control by descending precision signals.

According to this analysis, the possession of an apical dendrite reaching to layer 1 is a common characteristic of an error unit. In the study of the parvocellular relay through primate V1, referred to above (see Section 5), the possession of a tall apical dendritic tuft was one of the morphological characteristics distinguishing projection pyramids (presumed error units) from local pyramids (presumed expectation units) (Sawatari and Callaway, 2000). The large spiny stellate neurons of layer 4B that should serve as error units for the magnocellular pathway (projecting to V5) provide an exception to this principle; the lack of an apical dendrite in these cells is atypical, and interpreted as an adaptation to exclude parvocellular influence (Shipp and Zeki, 1989a; Sawatari and Callaway, 1996). Equally atypical, however, is the fact that layer 4B forms a discrete laminar target for direct backward input from V5 (Shipp and Zeki, 1989a; Rockland and Knutson, 2000) such that an apical dendrite is not needed to sample the putative precision signals. The deep layers are also worthy of consideration in this respect, as both layer 5 and layer 6 contain a significant, if minority proportion of forward-projecting neurons. One study compared the morphology of deep layer neurons in V2 projecting backward (to V1) or forward (to V4). The only cells shown to have tall apical dendrites stretching to layer 1 was a subset (44%) of forward-projecting layer 5 neurons (Markov et al., 2014). Whilst that may be a characteristic of error units, the remainder of forward-projecting cells in layer 5, and all those in layer 6 lacked such an appendage. If these cells were to receive precision signals, it might be provided by a deeper-terminating component of the back projection (but perhaps with a superficial origin – see **Figure 2**), or by an intrinsic relay from layer 2/3A to layer 5 (as mentioned above in consideration of communication with the pulvinar).

## 12. Summary

Perhaps the most basic assumption underlying the gPC template is the assignment of all pyramidal neurons to one of three categories of computational unit: expectation units encoding causes, error units encoding discrepancies between these expectations, and expected precision units encoding the reliability of the expectation; as above, these will hereon be referred to simply as expectation, error and precision units. How can we recognize these different categories? Although the physiological dynamics of each class would be expected to differ, in line with their different computational roles, these differences could be subtle, and likely to be subsumed within classification schemes based on more obvious characteristics relating to featural selectivity and tuning – in which expectation, error and precision units might conceivably share similar properties. To date, therefore, it is the pattern of extrinsic and intrinsic connectivity that offers better scope for distinguishing them. From the outset, predictive coding theories of cortical function have earmarked backward projecting neurons as prediction (expectation) units, and forward projecting neurons as error units (Mumford, 1992; Rao and Ballard, 1999; Friston, 2005). Because these two units perform distinct roles, it is a requirement of the theory that forward and backward projections are indeed sourced from two separate populations of neurons, and not to any great extent from neurons that project in both directions. As this proposition has only recently been verified (Berezovskii et al., 2011; Markov et al., 2014), it can be regarded as a successful anatomical prediction of predictive coding theory. However, as the gPC template shows, the equation of unit type and projection target can no longer be regarded as quite so simple. The following summarizes its microcircuitry by layer, noting elements that remain to be verified and elements that remain to be added.

### 12.1. Superficial Layers

In terms of extrinsic projections there are three classes of superficial pyramidal neuron: local (i.e., lacking an extrinsic projection)/expectation units, backward/precision units and forward/error units. In theory, expectation units with similar featural selectivity are networked together, and excite error units, that in turn excite precision units. The scheme as a whole remains conjectural owing to the inability of single, or even combined experimental techniques to address all the requisite criteria. Large field recordings of population activity through 2-photon imaging are now possible, and would be still more informative if coupled to retrograde labeling, or genetic marking of projection subtypes. A major limitation remains in the physiological characterisation of intrinsic network connectivity, as this requires paired (or multiple) intracellular recordings and so imposes a severe limitation on the number of cells whose contacts can be studied.

In addition, there are multiple classes of interneurons also forming specific connections with pyramidal cells and with each other. The gPC template shows a role for one type, fitting the known characteristics of the SST/Martinotti cell, but it is clear that the template omits much of the sophisticated neural dynamics of active cortex. For example, each of the three main classes of interneuron (PV, SST and VIP) has been shown to play a distinct role in mediating the top–down influence of cingulate cortex upon mouse V1 (Zhang et al., 2014). This projection terminates preferentially in layers 1 and 6, like backward connections from higher visual areas, and when optogenetically stimulated was found to mimic attention-like effects upon visual responses and enhanced performance of a visual discrimination task. Further investigation showed a center-surround organization, with VIP interneurons facilitating a central response of pyramidal neurons in layer 2/3 through inhibition of the other two classes, whilst SST interneurons contributed specifically to spatial surround suppression (Zhang et al., 2014). The extra relevance of these findings in the current context is that the gPC template identifies the superficial component of the back projection as a vehicle for precision, which in turn is allotted an agency in selective attention (Feldman and Friston, 2010).

Parvalbumin interneurons form the most frequent class of interneuron, with their own subclasses (Helm et al., 2013). A commonly ascribed function is gain control, as PV interneurons share the same excitatory contacts as pyramidal neurons, allowing for feedforward inhibition (Ma et al., 2010; Atallah et al., 2012; Wilson et al., 2012). PV neurons are often poorly tuned for visual features, but can mediate competitive interactions between better tuned pyramidal neurons by means of a networked divisive normalization mechanism (Wilson et al., 2012). Optogenetic activation of PV neurons in mouse V1 has been shown to enhance pyramidal cell tuning for orientation and direction, and improve behavioral performance (Lee et al., 2012). The gPC template, as configured here, illustrates the connectivity of a network representing just a single feature; however, rival networks of expectation units would be expected to compete in respect of mutual antagonism between opponent colors or directions, or indeed with respect to instances of bistable perception or binocular rivalry (Hohwy et al., 2008). A more sophisticated version of the gPC template could thus seek to incorporate a greater variety of interneuron functionality. Note that this would *not* include assigning interneurons the role of the principal computational units for ‘states’^4^ as indicated by Bastos et al. (2012). The idea is that the functional logic for nominating pyramidal neurons to represent causes applies equally to states, but the circuitry for the latter (that might involve extrinsic, lateral connections between areas of similar hierarchical level; Shipp et al., 2013) is also reserved for future consideration in an expanded gPC template.

### 12.2. Deep Layers

The deep layers likely also contain a mix of precision, error and expectation units, though only expectation units are specified by the gPC template as devised in **Figure 3**. There are two sub-types, one a local pyramid receiving descending predictions and the other a backward-projecting pyramid emitting descending predictions. The respective allocation of superficial and deep backward-projecting neurons exclusively to precision and expectation classes is possibly an over-simplification; either or both classes of extrinsic neuron could conceivably have a bilaminar distribution. It would be useful to know the specific laminar distribution of backward terminals originating from the superficial or deep layers, but even that would not necessarily prove decisive. Whilst the deep, backward-projecting pyramids tend to be concentrated in layer 6, there are also forward-projecting pyramids distributed more evenly between layers 5 and 6 (Markov et al., 2014). These are provisionally identified as error units, on account of the forward projection, but gPC lacks a computational rationale for the existence of a second class of error unit, prompting their exclusion from the current, minimalist template. If we follow the canonical microcircuit, we should expect separate relays linking to error units and expectation units from superficial units of either class, but details of specific network connectivity from superficial to deep are very limited. The same would hold for a third type of relay, conveying precision-related signals to layer 5, mooted above as source of input to the pulvinar (Kanai et al., 2015).

The distinction between layer 5 and layer 6 is better characterized anatomically by the separate populations of CS cells (projecting subcortically): layer 5 is the source of projections to many structures (thalamus, pons, tectum) whilst layer 6 has a separate set of smaller, CT cells with modulatory, as opposed to driving characteristics (Sherman and Guillery, 2011). Firm criteria for identification of precision, error and expectation units amongst CT and CS cells are yet to be established. Corticogeniculate cells should include a good proportion of expectation units, but there are a variety of subclasses (Briggs et al., 2016), so some might alternatively be precision units. If precision-related signals are conveyed to the pulvinar from layer 5, they should also reach the superior colliculus owing to axon collaterals (Guillery and Sherman, 2002). There is one (rather unique) class of corticotectal cell found at the layer 5/6 boundary of primate V1, the large so-called Meynert cell, that projects both to area V5/MT and the superior colliculus (but not to LGN) (Fries et al., 1985; Nhan and Callaway, 2012). If this were identified as an error unit, owing to the forward-going cortical projection, it would imply that error signals can also reach the superior colliculus, and potentially the pulvinar, following the colliculo-pulvinar projection.

## 12.3. Conclusion

Overall, predictive coding theories of brain function rationalize aspects of both perception and cortical structure. This is certainly a non-trivial achievement for a computational architecture with roots in information theory. This should not disguise the fact that the rather elementary gPC template discussed here fails to address much of the richness of cortical microcircuitry, whilst simultaneously specifying several circuit details that are yet to be verified. It is therefore at once suggestive of future avenues of investigation, falsifiable, and capable of evolution.

## Author Contributions

SS: manuscript and original graphics.

## Conflict of Interest Statement

The author declares that the research was conducted in the absence of any commercial or financial relationships that could be construed as a potential conflict of interest.

## Footnotes

1 This, older, method of *in vitro* photostimulation achieves non-selective neural activation by the use of a laser to unlock free glutamate from a chemically ‘caged’ form; by contrast, more recent optogenetic methods of photostimulation allow specific activation (or suppression) of subclasses of pyramidal cell or interneuron, and can be applied *in vivo* as well as *in vitro.*

2 NB. The arrangement shown in **Figure 3** (depicting a chain with one expectation unit receiving error input, a second making only reciprocal connections, and a third providing output to an error unit) is for diagrammatic convenience; a single superficial expectation unit can be envisaged to participate in all three (pathways 6, 5, and 13).

3 Katzel et al. (2011) further noted that the proportion, about 3:1, was similar to the relative frequency of simple and complex receptive fields recorded in layer 2/3 of mouse V1 (Niell and Stryker, 2008). The ‘simple/expectation’ and ‘complex/error’ associations implied by the comparison thereby concur with the analysis of the magnocellular relay through primate V1 (see Section 5).

4 i.e., expectation and error units for ‘states’ as opposed to ‘causes’, as distinguished in Section 3.

## References

[B1] Adams R. A. Shipp S. Friston K. J. (2013a). Predictions not commands: active inference in the motor system. *Brain Struct. Funct.* 218 611–643. 10.1007/s00429-012-0475-5 23129312 PMC3637647

[B2] Adams R. A. Stephan K. E. Brown H. R. Frith C. D. Friston K. J. (2013b). The computational anatomy of psychosis. *Front. Psychiatry* 4:47 10.3389/fpsyt.2013.00047 PMC3667557 23750138

[B3] Adesnik H. Bruns W. Taniguchi H. Huang Z. J. Scanziani M. (2012). A neural circuit for spatial summation in visual cortex. *Nature* 490 226–231. 10.1038/nature11526 23060193 PMC3621107

[B4] Anderson J. C. Martin K. A. C. (2006). Synaptic connection from cortical area V4 to V2 in macaque monkey. *J. Comp. Neurol.* 495 709–721. 10.1002/cne.20914 16506191

[B5] Angelucci A. Levitt J. B. Walton E. J. Hupe J. M. Bullier J. Lund J. S. (2002). Circuits for local and global signal integration in primary visual cortex. *J. Neurosci.* 22 8633–8646. 12351737 10.1523/JNEUROSCI.22-19-08633.2002 PMC6757772

[B6] Atallah B. V. Bruns W. Carandini M. Scanziani M. (2012). Parvalbumin-expressing interneurons linearly transform cortical responses to visual stimuli. *Neuron* 73 159–170. 10.1016/j.neuron.2011.12.013 22243754 PMC3743079

[B7] Barone P. Batardiere A. Knoblauch K. Kennedy H. (2000). Laminar distribution of neurons in extrastriate areas projecting to visual areas V1 and V4 correlates with the hierarchical rank and indicates the operation of a distance rule. *J. Neurosci.* 20 3263–32681. 10777791 10.1523/JNEUROSCI.20-09-03263.2000 PMC6773101

[B8] Bastos A. M. Usrey W. M. Adams R. A. Mangun G. R. Fries P. Friston K. J. (2012). Canonical microcircuits for predictive coding. *Neuron* 76 695–711. 10.1016/j.neuron.2012.10.038 23177956 PMC3777738

[B9] Berezovskii V. K. Nassi J. J. Born R. T. (2011). Segregation of feedforward and feedback projections in mouse visual cortex. *J. Comp. Neurol.* 519 3672–3683. 10.1002/cne.22675 21618232 PMC3219532

[B10] Beul S. F. Hilgetag C. C. (2015). Towards a “canonical” agranular cortical microcircuit. *Front. Neuroanat.* 8:165 10.3389/fnana.2014.00165 PMC4294159 25642171

[B11] Blasdel G. G. Fitzpatrick D. (1984). Physiological organization of layer 4 in macaque striate cortex. *J. Neurosci.* 4 880–895. 6200586 10.1523/JNEUROSCI.04-03-00880.1984 PMC6564839

[B12] Bonin V. Histed M. H. Yurgenson S. Reid R. C. (2011). Local diversity and fine-scale organization of receptive fields in mouse visual cortex. *J. Neurosci.* 31 18506–18521. 10.1523/jneurosci.2974-11.2011 22171051 PMC3758577

[B13] Bortone D. S. Olsen S. R. Scanziani M. (2014). Translaminar inhibitory cells recruited by layer 6 corticothalamic neurons suppress visual cortex. *Neuron* 82 474–485. 10.1016/j.neuron.2014.02.021 24656931 PMC4068343

[B14] Bosking W. H. Zhang Y. Schofield B. Fitzpatrick D. (1997). Orientation selectivity and the arrangement of horizontal connections in tree shrew striate cortex. *J. Neurosci.* 17 2112–2127. 9045738 10.1523/JNEUROSCI.17-06-02112.1997 PMC6793759

[B15] Briggs F. Callaway E. M. (2001). Layer-specific input to distinct cell types in layer 6 of monkey primary visual cortex. *J. Neurosci.* 21 3600–3608. 11331389 10.1523/JNEUROSCI.21-10-03600.2001 PMC1820845

[B16] Briggs F. Callaway E. M. (2005). Laminar patterns of local excitatory input to layer 5 neurons in macaque primary visual cortex. *Cereb. Cortex* 15 479–488. 10.1093/cercor/bhh154 15319309 PMC1820846

[B17] Briggs F. Kiley C. W. Callaway E. M. Usrey W. M. (2016). Morphological substrates for parallel streams of corticogeniculate feedback originating in both V1 and V2 of the macaque monkey. *Neuron* 90 388–399. 10.1016/j.neuron.2016.02.038 27041497 PMC4840039

[B18] Briggs F. Usrey W. M. (2007). A fast, reciprocal pathway between the lateral geniculate nucleus and visual cortex in the macaque monkey. *J. Neurosci.* 27 5431–5436. 10.1523/JNEUROSCI.1035-07.2007 17507565 PMC2888515

[B19] Briggs F. Usrey W. M. (2009). Parallel processing in the corticogeniculate pathway of the macaque monkey. *Neuron* 62 135–146. 10.1016/j.neuron.2009.02.024 19376073 PMC2789995

[B20] Briggs F. Usrey W. M. (2011). Corticogeniculate feedback and visual processing in the primate. *J. Physiol. (Lond).* 589 33–40. 10.1113/jphysiol.2010.193599 20724361 PMC3039257

[B21] Bullier J. Kennedy H. (1987). Axonal bifurcation in the visual system. *Trends Neurosci.* 10 205–210. 10.1016/0166-2236(87)90152-4

[B22] Cauller L. (1995). Layer I of primary sensory neocortex: where top-down converges upon bottom-up. *Behav. Brain Res.* 71 163–170. 10.1016/0166-4328(95)00032-1 8747184

[B23] Cauller L. J. Clancy B. Connors B. W. (1998). Backward cortical projections to primary somatosensory cortex in rats extend long horizontal axons in layer I. *J. Comp. Neurol.* 390 297–310. 10.1002/(SICI)1096-9861(19980112)390:2<297::AID-CNE11>3.0.CO;2-V 9453672

[B24] Chu Z. Galarreta M. Hestrin S. (2003). Synaptic interactions of late-spiking neocortical neurons in layer 1. *J. Neurosci.* 23 96–102. 12514205 10.1523/JNEUROSCI.23-01-00096.2003 PMC6742162

[B25] Coogan T. A. Burkhalter A. (1993). Hierarchical organization of areas in rat visual cortex. *J. Neurosci.* 13 3749–3772. 7690066 10.1523/JNEUROSCI.13-09-03749.1993 PMC6576464

[B26] Cossell L. Iacaruso M. F. Muir D. R. Houlton R. Sader E. N. Ko H. (2015). Functional organization of excitatory synaptic strength in primary visual cortex. *Nature* 518 399–403. 10.1038/nature14182 25652823 PMC4843963

[B27] Cudeiro J. Sillito A. M. (2006). Looking back: corticothalamic feedback and early visual processing. *Trends Neurosci.* 29 298–306. 10.1016/j.tins.2006.05.002 16712965

[B28] Dantzker J. L. Callaway E. M. (2000). Laminar sources of synaptic input to cortical inhibitory interneurons and pyramidal neurons. *Nat. Neurosci.* 3 701–707. 10.1038/76656 10862703

[B29] Dayan P. Hinton G. E. Neal R. M. Zemel R. S. (1995). The Helmholtz machine. *Neural Comput.* 7 889–904. 10.1162/neco.1995.7.5.889 7584891

[B30] Dehay C. Kennedy H. Kosik K. S. (2015). The outer subventricular zone and primate-specific cortical complexification. *Neuron* 85 683–694. 10.1016/j.neuron.2014.12.060 25695268

[B31] Douglas R. J. Martin K. A. (1991). A functional microcircuit for cat visual cortex. *J. Physiol. (Lond).* 440 735–769. 10.1113/jphysiol.1991.sp018733 1666655 PMC1180177

[B32] Feldman H. Friston K. J. (2010). Attention, uncertainty, and free-energy. *Front. Hum. Neurosci.* 4:215 10.3389/fnhum.2010.00215 PMC3001758 21160551

[B33] Felleman D. J. Van Essen D. C. (1991). Distributed hierarchical processing in the primate cerebral cortex. *Cereb. Cortex* 1 1–47. 10.1093/cercor/1.1.1 1822724

[B34] Fishell G. Rudy B. (2012). Mechanisms of inhibition within the telencephalon: “where the wild things are”. *Annu. Rev. Neurosci.* 34 535–567. 10.1146/annurev-neuro-061010-113717 PMC3556485 21469958

[B35] Freiwald W. A. Tsao D. Y. (2010). Functional compartmentalization and viewpoint generalization within the macaque face-processing system. *Science* 330 845–851. 10.1126/science.1194908 21051642 PMC3181095

[B36] Fries W. Keizer K. Kuypers H. G. J. M. (1985). Large layer VI cells in macaque striate cortex (Meynert cells) project to both superior colliculus and prestriate visual area V5. *Exp. Brain Res.* 58 613–616. 10.1007/BF00235878 3839191

[B37] Friston K. (2005). A theory of cortical responses. *Philos. Trans. R. Soc. Lond. B. Biol. Sci.* 360 815–836. 10.1098/rstb.2005.1622 15937014 PMC1569488

[B38] Friston K. (2008). Hierarchical models in the brain. *PLoS Comput. Biol.* 4:e1000211 10.1371/journal.pcbi.1000211 PMC2570625 18989391

[B39] Friston K. (2009). The free-energy principle: a rough guide to the brain? *Trends Cogn. Sci.* 13 293–301. 10.1016/j.tics.2009.04.005 19559644

[B40] Friston K. (2010). The free-energy principle: a unified brain theory? *Nat. Rev. Neurosci.* 11 127–138. 10.1038/nrn2787 20068583

[B41] Friston K. Kiebel S. (2009). Predictive coding under the free-energy principle. *Philos. Trans. R. Soc. Lond. B Biol. Sci.* 364 1211–1221. 10.1098/rstb.2008.0300 19528002 PMC2666703

[B42] Friston K. Mattout J. Kilner J. (2011). Action understanding and active inference. *Biol. Cybern.* 104 137–160. 10.1007/s00422-011-0424-z 21327826 PMC3491875

[B43] Fujita I. Fujita T. (1996). Intrinsic connections in the macaque inferior temporal cortex. *J. Comp. Neurol.* 368 467–486. 10.1002/(SICI)1096-9861(19960513)368:4<467::AID-CNE1>3.0.CO;2-2 8744437

[B44] Ghanem A. Conzelmann K. K. (2016). G gene-deficient single-round rabies viruses for neuronal circuit analysis. *Virus Res.* 216 41–54. 10.1016/j.virusres.2015.05.023 26065596

[B45] Gilbert C. D. (1983). Microcircuitry of the visual cortex. *Annu. Rev. Neurosci.* 6 217–247. 10.1146/annurev.ne.06.030183.001245 6132585

[B46] Godlove D. C. Maier A. Woodman G. F. Schall J. D. (2014). Microcircuitry of agranular frontal cortex: testing the generality of the canonical cortical microcircuit. *J. Neurosci.* 34 5355–5369. 10.1523/jneurosci.5127-13.2014 24719113 PMC3983808

[B47] Gonchar Y. Burkhalter A. (1999). Differential subcellular localization of forward and feedback interareal inputs to parvalbumin expressing GABAergic neurons in rat visual cortex. *J. Comp. Neurol.* 406 346–360. 10.1002/(SICI)1096-9861(19990412)406:3<346::AID-CNE4>3.0.CO;2-E 10102500

[B48] Gonchar Y. Burkhalter A. (2003). Distinct GABAergic targets of feedforward and feedback connections between lower and higher areas of rat visual cortex. *J. Neurosci.* 23 10904–10912. 14645486 10.1523/JNEUROSCI.23-34-10904.2003 PMC6740993

[B49] Gregory R. L. (1980). Perceptions as hypotheses. *Philos. Trans. R. Soc. Lond. B Biol. Sci.* 290 181–197. 10.1098/rstb.1980.0090 6106237

[B50] Grieve K. L. Sillito A. M. (1995). Differential properties of cells in the feline primary visual cortex providing the corticofugal feedback to the lateral geniculate nucleus and visual claustrum. *J. Neurosci.* 15 4868–4874. 7623117 10.1523/JNEUROSCI.15-07-04868.1995 PMC6577860

[B51] Groh A. Meyer H. S. Schmidt E. F. Heintz N. Sakmann B. Krieger P. (2010). Cell-type specific properties of pyramidal neurons in neocortex underlying a layout that Is modifiable depending on the cortical area. *Cereb. Cortex* 20 826–836. 10.1093/cercor/bhp152 19643810

[B52] Guillery R. W. Sherman S. M. (2002). The thalamus as a monitor of motor outputs. *Philos. Trans. R. Soc. Lond. B* 357 1809–1821. 10.1098/rstb.2002.1171 12626014 PMC1693090

[B53] Gur M. Kagan I. Snodderly D. M. (2005). Orientation and direction selectivity of neurons in V1 of alert monkeys: functional relationships and laminar distributions. *Cereb. Cortex* 15 1207–1221. 10.1093/cercor/bhi003 15616136

[B54] Hawken M. J. Parker A. J. Lund J. S. (1988). Laminar organization and contrast selectivity of direction selective cells in the striate cortex of the Old-World monkey. *J. Neurosci.* 8 3541–3548. 3193169 10.1523/JNEUROSCI.08-10-03541.1988 PMC6569616

[B55] Helm J. Akgul G. Wollmuth L. P. (2013). Subgroups of parvalbumin-expressing interneurons in layers 2/3 of the visual cortex. *J. Neurophysiol.* 109 1600–1613. 10.1152/jn.00782.2012 23274311 PMC3602937

[B56] Helmholtz H. (1860/1962). “Handbuch der physiologischen optik,” in *English Translation* Vol. 3 ed. Southall J. P. C. (New York, NY: Dover).

[B57] Henry G. H. Salin P. A. Bullier J. (1991). Projections from areas 18 and 19 to cat striate cortex: divergence and laminar specificity. *Eur. J. Neurosci.* 3 186–200. 10.1111/j.1460-9568.1991.tb00079.x 12106217

[B58] Hohwy J. Roepstorff A. Friston K. (2008). Predictive coding explains binocular rivalry: an epistemological review. *Cognition* 108 687–701. 10.1016/j.cognition.2008.05.010 18649876

[B59] Hooks B. M. Hires S. A. Zhang Y. X. Huber D. Petreanu L. Svoboda K. (2011). Laminar analysis of excitatory local circuits in vibrissal motor and sensory cortical areas. *PLoS Biol.* 9:e1000572 10.1371/journal.pbio.1000572 PMC3014926 21245906

[B60] Hubel D. H. Wiesel T. N. (1962). Receptive fields, binocular interaction and functional architecture in the cat’s visual cortex. *J. Physiol. (Lond).* 160 106–154. 10.1113/jphysiol.1962.sp006837 14449617 PMC1359523

[B61] Hutsler J. J. Lee D. G. Porter K. K. (2005). Comparative analysis of cortical layering and supragranular layer enlargement in rodent carnivore and primate species. *Brain Res.* 1052 71–81. 10.1016/j.brainres.2005.06.015 16018988

[B62] Jehee J. F. Ballard D. H. (2009). Predictive feedback can account for biphasic responses in the lateral geniculate nucleus. *PLoS Comput. Biol.* 5:e1000373 10.1371/journal.pcbi.1000373 PMC2670540 19412529

[B63] Jiang X. Wang G. Lee A. J. Stornetta R. L. Zhu J. J. (2013). The organization of two new cortical interneuronal circuits. *Nat. Neurosci.* 16 210–218. 10.1038/nn.3305 23313910 PMC3589105

[B64] Johnson R. R. Burkhalter A. (1994). Evidence for excitatory amino acid neurotransmitters in forward and feedback corticocortical pathways within rat visual cortex. *Eur. J. Neurosci.* 6 272–286. 10.1111/j.1460-9568.1994.tb00270.x 7513241

[B65] Kageyama G. H. Gallivan M. E. Gallardo K. A. Robertson R. T. (1990). Relationships between patterns of acetylcholinesterase activity and geniculocortical terminal fields in developing and mature rat visual cortex. *Brain Res. Dev. Brain Res.* 53 139–144. 10.1016/0165-3806(90)90135-L 1693552

[B66] Kampa B. M. Letzkus J. J. Stuart G. J. (2006). Cortical feed-forward networks for binding different streams of sensory information. *Nat. Neurosci.* 9 1472–1473. 10.1038/nn1798 17099707

[B67] Kanai R. Komura Y. Shipp S. Friston K. (2015). Cerebral hierarchies: predictive processing, precision and the pulvinar. *Philos. Trans. R. Soc. Lond. B Biol. Sci.* 370:20140169 10.1098/rstb.2014.0169 PMC4387510 25823866

[B68] Kanizsa G. (1979). *Organization in Vision.* New York, NY: Praeger Publishing.

[B69] Katzel D. Zemelman B. V. Buetfering C. Wolfel M. Miesenbock G. (2011). The columnar and laminar organization of inhibitory connections to neocortical excitatory cells. *Nat. Neurosci.* 14 100–107. 10.1038/nn.2687 21076426 PMC3011044

[B70] Kim E. J. Juavinett A. L. Kyubwa E. M. Jacobs M. W. Callaway E. M. (2015). Three types of cortical layer 5 neurons that differ in brain-wide connectivity and function. *Neuron* 88 1253–1267. 10.1016/j.neuron.2015.11.002 26671462 PMC4688126

[B71] Knill D. C. Pouget A. (2004). The Bayesian brain: the role of uncertainty in neural coding and computation. *Trends Neurosci.* 27 712–719. 10.1016/j.tins.2004.10.007 15541511

[B72] Ko H. Hofer S. B. Pichler B. Buchanan K. A. Sjostrom P. J. Mrsic-Flogel T. D. (2011). Functional specificity of local synaptic connections in neocortical networks. *Nature* 473 87–91. 10.1038/nature09880 21478872 PMC3089591

[B73] Kogo N. Trengove C. (2015). Is predictive coding theory articulated enough to be testable? *Front. Comput. Neurosci.* 9:111 10.3389/fncom.2015.00111 PMC4561670 26441621

[B74] Kondo S. Ohki K. (2016). Laminar differences in the orientation selectivity of geniculate afferents in mouse primary visual cortex. *Nat. Neurosci.* 19 316–319. 10.1038/nn.4215 26691830

[B75] Larkum M. (2013). A cellular mechanism for cortical associations: an organizing principle for the cerebral cortex. *Trends Neurosci.* 36 141–151. 10.1016/j.tins.2012.11.006 23273272

[B76] Lawson R. P. Rees G. Friston K. J. (2014). An aberrant precision account of autism. *Front. Hum. Neurosci.* 8:302 10.3389/fnhum.2014.00302 PMC4030191 24860482

[B77] Lee S. H. Kwan A. C. Zhang S. Phoumthipphavong V. Flannery J. G. Masmanidis S. C. (2012). Activation of specific interneurons improves V1 feature selectivity and visual perception. *Nature* 488 379–383. 10.1038/nature11312 22878719 PMC3422431

[B78] Lee T. S. Mumford D. (2003). Hierarchical Bayesian inference in the visual cortex. *J. Opt. Soc. Am. A Opt. Image Sci. Vis.* 20 1434–1448. 10.1364/JOSAA.20.001434 12868647

[B79] Lee W. C. A. Bonin V. Reed M. Graham B. J. Hood G. Glattfelder K. (2016). Anatomy and function of an excitatory network in the visual cortex. *Nature* 532 370–374. 10.1038/nature17192 27018655 PMC4844839

[B80] Letzkus J. J. Wolff S. B. Meyer E. M. Tovote P. Courtin J. Herry C. (2011). A disinhibitory microcircuit for associative fear learning in the auditory cortex. *Nature* 480 331–335. 10.1038/nature10674 22158104

[B81] Li Y. T. Ibrahim L. A. Liu B. H. Zhang L. I. Tao H. W. (2013). Linear transformation of thalamocortical input by intracortical excitation. *Nat. Neurosci.* 16 1324–1330. 10.1038/nn.3494 23933750 PMC3855439

[B82] Lien A. D. Scanziani M. (2013). Tuned thalamic excitation is amplified by visual cortical circuits. *Nat. Neurosci.* 16 1315–1323. 10.1038/nn.3488 23933748 PMC3774518

[B83] Livingstone M. S. Hubel D. H. (1984a). Anatomy and physiology of a color system in the primate visual cortex. *J. Neurosci.* 4 309–356. 6198495 10.1523/JNEUROSCI.04-01-00309.1984 PMC6564760

[B84] Livingstone M. S. Hubel D. H. (1984b). Specificity of intrinsic connections in primate primary visual cortex. *J. Neurosci.* 4 2830–2835. 6209365 10.1523/JNEUROSCI.04-11-02830.1984 PMC6564722

[B85] Llano D. A. Sherman S. M. (2009). Differences in intrinsic properties and local network connectivity of Iidentified layer 5 and layer 6 adult mouse auditory corticothalamic neurons support a dual corticothalamic projection hypothesis. *Cereb. Cortex* 19 2810–2826. 10.1093/cercor/bhp050 19351905 PMC2774389

[B86] Lund J. S. Hendrickson A. E. Ogren M. P. Tobin E. A. (1981). Anatomical organization of primate visual cortex area VII. *J. Comp. Neurol.* 202 19–45. 10.1002/cne.902020104 6793644

[B87] Ma W. P. Liu B. H. Li Y. T. Huang Z. J. Zhang L. I. Tao H. W. (2010). Visual representations by cortical somatostatin inhibitory neurons–selective but with weak and delayed responses. *J. Neurosci.* 30 14371–14379. 10.1523/jneurosci.3248-10.2010 20980594 PMC3001391

[B88] Malach R. Amir Y. Harel M. Grinvald A. (1993). Relationship between intrinsic connections and functional architecture revealed by optical imaging and in vivo targeted biocytin injections in primate striate cortex. *Proc. Natl. Acad. Sci. U.S.A.* 90 10469–10473. 10.1073/pnas.90.22.10469 8248133 PMC47798

[B89] Markov N. T. Vezoli J. Chameau P. Falchier A. Quilodran R. Huissoud C. (2014). Anatomy of hierarchy: feedforward and feedback pathways in macaque visual cortex. *J. Comp. Neurol.* 522 225–259. 10.1002/cne.23458 23983048 PMC4255240

[B90] Markram H. Muller E. Ramaswamy S. Reimann M. W. Abdellah M. Sanchez C. A. (2015). Reconstruction and simulation of neocortical microcircuitry. *Cell* 163 456–492. 10.1016/j.cell.2015.09.029 26451489

[B91] Maunsell J. H. R. Van Essen D. C. (1983). The connections of the middle temporal area and their relationship to a cortical hierarchy in the macaque monkey. *J. Neurosci.* 3 2563–2586. 6655500 10.1523/JNEUROSCI.03-12-02563.1983 PMC6564662

[B92] Morgenstern N. A. Bourg J. Petreanu L. (2016). Multilaminar networks of cortical neurons integrate common inputs from sensory thalamus. *Nat. Neurosci.* 19 1034–1040. 10.1038/nn.4339 27376765

[B93] Movshon J. A. Newsome W. T. (1996). Visual response properties of striate cortical neurons projecting to area MT in macaque monkeys. *J. Neurosci.* 16 7733–7741. 8922429 10.1523/JNEUROSCI.16-23-07733.1996 PMC6579106

[B94] Mumford D. (1992). On the computational architecture of the neocortex. II. The role of cortico-cortical loops. *Biol. Cybern.* 66 241–251. 1540675 10.1007/BF00198477

[B95] Murphy P. C. Duckett S. G. Sillito A. M. (1999). Feedback connections to the lateral geniculate nucleus and cortical response properties. *Science* 286 1552–1554. 10.1126/science.286.5444.1552 10567260

[B96] Nassi J. J. Callaway E. M. (2006). Multiple circuits relaying primate parallel visual pathways to the middle temporal area. *J. Neurosci.* 26 12789–12798. 10.1523/JNEUROSCI.4044-06.2006 17151282 PMC2629496

[B97] Nassi J. J. Callaway E. M. (2007). Specialized circuits from primary visual cortex to V2 and area MT. *Neuron* 55 799–808. 10.1016/j.neuron.2007.07.037 17785186 PMC2727861

[B98] Nassi J. J. Cepko C. L. Born R. T. Beier K. T. (2015). Neuroanatomy goes viral! *Front. Neuroanat.* 9:80 10.3389/fnana.2015.00080 PMC4486834 26190977

[B99] Nhan H. L. Callaway E. M. (2012). Morphology of superior colliculus- and middle temporal area-projecting neurons in primate primary visual cortex. *J. Comp. Neurol.* 520 52–80. 10.1002/cne.22685 21674487 PMC3886567

[B100] Niell C. M. Stryker M. P. (2008). Highly selective receptive fields in mouse visual cortex. *J. Neurosci.* 28 7520–7536. 10.1523/jneurosci.0623-08.2008 18650330 PMC3040721

[B101] Nienborg H. Hasenstaub A. Nauhaus I. Taniguchi H. Huang Z. J. Callaway E. M. (2013). Contrast dependence and differential contributions from somatostatin- and parvalbumin-expressing neurons to spatial integration in mouse V1. *J. Neurosci.* 33 11145–11154. 10.1523/jneurosci.5320-12.2013 23825418 PMC3718383

[B102] Ninomiya T. Dougherty K. Godlove D. C. Schall J. D. Maier A. (2015). Microcircuitry of agranular frontal cortex: contrasting laminar connectivity between occipital and frontal areas. *J. Neurophysiol.* 113 3242–3255. 10.1152/jn.00624.2014 25744881 PMC4440241

[B103] Ninomiya T. Sawamura H. Inoue K. Takada M. (2011). Differential architecture of multisynaptic geniculo-cortical pathways to V4 and MT. *Cereb. Cortex* 21 2797–2808. 10.1093/cercor/bhr078 21515714

[B104] Ohki K. Chung S. Ch’ng Y. H. Kara P. Reid R. C. (2005). Functional imaging with cellular resolution reveals precise micro-architecture in visual cortex. *Nature* 433 597–603. 10.1038/nature03274 15660108

[B105] Olsen S. R. Bortone D. S. Adesnik H. Scanziani M. (2012). Gain control by layer six in cortical circuits of vision. *Nature* 483 47–52. 10.1038/nature10835 22367547 PMC3636977

[B106] Perin R. Berger T. K. Markram H. (2011). A synaptic organizing principle for cortical neuronal groups. *Proc. Natl. Acad. Sci. U.S.A.* 108 5419–5424. 10.1073/pnas.1016051108 21383177 PMC3069183

[B107] Perkel D. J. Bullier J. Kennedy H. (1986). Topography of the afferent connectivity of area 17 in the macaque monkey: a double-labelling study. *J. Comp. Neurol.* 253 374–402. 10.1002/cne.902530307 3793996

[B108] Petrof I. Viaene A. N. Sherman S. M. (2012). Two populations of corticothalamic and interareal corticocortical cells in the subgranular layers of the mouse primary sensory cortices. *J. Comp. Neurol.* 520 1678–1686. 10.1002/cne.23006 22120996 PMC3561675

[B109] Pfeffer C. K. Xue M. He M. Huang Z. J. Scanziani M. (2013). Inhibition of inhibition in visual cortex: the logic of connections between molecularly distinct interneurons. *Nat. Neurosci.* 16 1068–1076. 10.1038/nn.3446 23817549 PMC3729586

[B110] Pi H. J. Hangya B. Kvitsiani D. Sanders J. I. Huang Z. J. Kepecs A. (2013). Cortical interneurons that specialize in disinhibitory control. *Nature* 503 521–524. 10.1038/nature12676 24097352 PMC4017628

[B111] Piscopo D. M. El-Danaf R. N. Huberman A. D. Niell C. M. (2013). Diverse visual features encoded in mouse lateral geniculate nucleus. *J. Neurosci.* 33 4642–4656. 10.1523/jneurosci.5187-12.2013 23486939 PMC3665609

[B112] Pollen D. A. (2008). Fundamental requirements for primary visual perception. *Cereb. Cortex* 18 1991–1998. 10.1093/cercor/bhm226 18089579

[B113] Poort J. Raudies F. Wannig A. Lamme V. A. Neumann H. Roelfsema P. R. (2012). The role of attention in figure-ground segregation in areas V1 and V4 of the visual cortex. *Neuron* 75 143–156. 10.1016/j.neuron.2012.04.032 22794268

[B114] Purushothaman G. Marion R. Li K. Casagrande V. A. (2012). Gating and control of primary visual cortex by pulvinar. *Nat. Neurosci.* 15 905–912. 10.1038/nn.3106 22561455 PMC3430824

[B115] Qiu F. T. Sugihara T. der Heydt R. (2007). Figure-ground mechanisms provide structure for selective attention. *Nat. Neurosci.* 10 1492–1499. 10.1038/nn1989 17922006 PMC2666969

[B116] Rao R. P. Ballard D. H. (1999). Predictive coding in the visual cortex: a functional interpretation of some extra-classical receptive-field effects. *Nat. Neurosci.* 2 79–87. 10.1038/4580 10195184

[B117] Rockland K. S. Drash G. W. (1996). Collateralized divergent feedback connections that target multiple cortical areas. *J. Comp. Neurol.* 373 529–548. 10.1002/(SICI)1096-9861(19960930)373:4<529::AID-CNE5>3.0.CO;2-3 8889943

[B118] Rockland K. S. Knutson T. (2000). Feedback connections from area MT of the squirrel monkey to areas V1 and V2. *J. Comp. Neurol.* 425 345–368. 10.1002/1096-9861(20000925)425:3<345::AID-CNE2>3.0.CO;2-O 10972937

[B119] Rockland K. S. Pandya D. N. (1979). Laminar origins and terminations of cortical connections of the occipital lobe in the rhesus monkey. *Brain Res.* 179 3–20. 10.1016/0006-8993(79)90485-2 116716

[B120] Rockland K. S. Saleem K. S. Tanaka K. (1994). Divergent feedback connections from areas V4 and TEO in the macaque. *Vis. Neurosci.* 11 579–600. 10.1017/S0952523800002480 8038130

[B121] Rockland K. S. Van Hoesen G. W. (1994). Direct temporal-occipital feedback connections to striate cortex (V1) in the macaque monkey. *Cereb. Cortex* 4 300–313. 10.1093/cercor/4.3.300 8075534

[B122] Rockland K. S. Virga A. (1990). Organization of individual cortical axons projecting from area V1 (area 17) to V2 (area 18) in the macaque monkey. *Vis. Neurosci.* 4 1–28. 10.1017/S095252380000273X 2176095

[B123] Rolls E. T. (1992). Neurophysiological mechanisms underlying face processing within and beyond the temporal cortical visual areas. *Philos. Trans. R. Soc. Lond. B Biol. Sci.* 335 11–20. 10.1098/rstb.1992.0002 1348130

[B124] Saalmann Y. B. Pinsk M. A. Wang L. Li X. Kastner S. (2012). The pulvinar regulates information transmission between cortical areas based on attention demands. *Science* 337 753–756. 10.1126/science.1223082 22879517 PMC3714098

[B125] Sawatari A. Callaway E. M. (1996). Convergence of magno-and parvocellular pathways in layer 4B of macaque primary visual cortex. *Nature* 380 442–446. 10.1038/380442a0 8602243

[B126] Sawatari A. Callaway E. M. (2000). Diversity and cell type specificity of local excitatory connections to neurons in layer 3B of monkey primary visual cortex. *Neuron* 25 459–471. 10.1016/S0896-6273(00)80908-3 10719899

[B127] Schwartenbeck P. FitzGerald T. H. B. Mathys C. Dolan R. Friston K. (2015). The dopaminergic midbrain encodes the expected certainty about desired outcomes. *Cereb. Cortex* 25 3434–3445. 10.1093/cercor/bhu159 25056572 PMC4585497

[B128] Shapley R. Hawken M. J. (2011). Color in the Cortex: single- and double-opponent cells. *Vision Res.* 51 701–717. 10.1016/j.visres.2011.02.012 21333672 PMC3121536

[B129] Sherman S. M. Guillery R. W. (1996). Functional organization of thalamocortical relays. *J. Neurophysiol.* 76 1367–1395. 8890259 10.1152/jn.1996.76.3.1367

[B130] Sherman S. M. Guillery R. W. (2011). Distinct functions for direct and transthalamic corticocortical connections. *J. Neurophysiol.* 106 1068–1077. 10.1152/jn.00429.2011 21676936

[B131] Shipp S. (2003). The functional logic of cortico-pulvinar connections. *Philos. Trans. R. Soc. Lond. B. Biol. Sci.* 358 1605–1624. 10.1098/rstb.2002.1213 14561322 PMC1693262

[B132] Shipp S. (2007). Structure and function of the cerebral cortex. *Curr. Biol.* 17 443–449. 10.1016/j.cub.2007.03.044 17580069

[B133] Shipp S. Adams R. A. Friston K. J. (2013). Reflections on agranular architecture: predictive coding in the motor cortex. *Trends Neurosci.* 36 706–716. 10.1016/j.tins.2013.09.004 24157198 PMC3858810

[B134] Shipp S. Zeki S. (1989a). The organization of connections between areas V5 and V1 in macaque monkey visual cortex. *Eur. J. Neurosci.* 1 309–332. 10.1111/j.1460-9568.1989.tb00798.x 12106142

[B135] Shipp S. Zeki S. (1989b). The organization of connections between areas V5 and V2 in macaque monkey visual cortex. *Eur. J. Neurosci.* 1 333–354. 10.1111/j.1460-9568.1989.tb00798.x 12106143

[B136] Shlosberg D. Amitai Y. Azouz R. (2006). Time-dependent, layer-specific modulation of sensory responses mediated by neocortical layer 1. *J. Neurophysiol.* 96 3170–3182. 10.1152/jn.00520.2006 17110738

[B137] Song S. Sjostrom P. J. Reigl M. Nelson S. Chklovskii D. B. (2005). Highly nonrandom features of synaptic connectivity in local cortical circuits. *PLoS Biol.* 3:e68 10.1371/journal.pbio.0030068 PMC1054880 15737062

[B138] Sousa A. P. Pinon M. C. Gattass R. Rosa M. G. (1991). Topographic organization of cortical input to striate cortex in the Cebus monkey: a fluorescent tracer study. *J. Comp. Neurol.* 308 665–682. 10.1002/cne.903080411 1865021

[B139] Spratling M. W. (2016). A review of predictive coding algorithms. *Brain Cogn.* 10.1016/j.bandc.2015.11.003 [Epub ahead of print]. 26809759

[B140] Stettler D. D. Das A. Bennett J. Gilbert C. D. (2002). Lateral connectivity and contextual interactions in macaque primary visual cortex. *Neuron* 36 739–750. 10.1016/S0896-6273(02)01029-2 12441061

[B141] Sun W. Z. Tan Z. C. Mensh B. D. Ji N. (2016). Thalamus provides layer 4 of primary visual cortex with orientation- and direction-tuned inputs. *Nat. Neurosci.* 19 308–315. 10.1038/nn.4196 26691829 PMC4731241

[B142] Tang J. Y. Jimenez S. C. A. Chakraborty S. Schultz S. R. (2016). Visual receptive field properties of neurons in the mouse lateral geniculate nucleus. *PLoS ONE* 11:34 10.1371/journal.pone.0146017 PMC4712148 26741374

[B143] Tasic B. Menon V. Nguyen T. N. Kim T. K. Jarsky T. Yao Z. (2016). Adult mouse cortical cell taxonomy revealed by single cell transcriptomics. *Nat. Neurosci.* 19 335–346. 10.1038/nn.4216 26727548 PMC4985242

[B144] Thomson A. M. (2010). Neocortical layer 6, a review. *Front. Neuroanat.* 4:13 10.3389/fnana.2010.00013 PMC2885865 20556241

[B145] Thomson A. M. Bannister A. P. (2003). Interlaminar connections in the neocortex. *Cereb. Cortex* 13 5–14. 10.1093/cercor/13.1.5 12466210

[B146] Thomson A. M. Lamy C. (2007). Functional maps of neocortical local circuitry. *Front. Neurosci.* 1:19–42. 10.3389/neuro.01.1.1.002.2007 18982117 PMC2518047

[B147] Thomson A. M. West D. C. Wang Y. Bannister A. P. (2002). Synaptic connections and small circuits involving excitatory and inhibitory neurons in layers 2-5 of adult rat and cat neocortex: triple intracellular recordings and biocytin labelling in vitro. *Cereb. Cortex* 12 936–953. 10.1093/cercor/12.9.936 12183393

[B148] Valverde F. (1978). The organization of area 18 in the monkey. A Golgi study. *Anat. Embryol. (Berl).* 154 305–334. 10.1007/BF00345659 101094

[B149] Van Hooser S. D. Heimel J. A. Chung S. Nelson S. B. (2006). Lack of patchy horizontal connectivity in primary visual cortex of a mammal without orientation maps. *J. Neurosci.* 26 7680–7692. 10.1523/jneurosci.0108-06.2006 16855096 PMC6674269

[B150] Van Hooser S. D. Heimel J. A. Chung S. Nelson S. B. Toth L. J. (2005). Orientation selectivity without orientation maps in visual cortex of a highly visual mammal. *J. Neurosci.* 25 19–28. 10.1523/jneurosci.4042-04.2005 15634763 PMC6725193

[B151] Velez-Fort M. Rousseau C. V. Niedworok C. J. Wickersham I. R. Rancz E. A. Brown A. P. (2014). The stimulus selectivity and connectivity of layer six principal cells reveals cortical microcircuits underlying visual processing. *Neuron* 83 1431–1443. 10.1016/j.neuron.2014.08.001 25175879 PMC4175007

[B152] Wang W. Jones H. E. Andolina I. M. Salt T. E. Sillito A. M. (2006). Functional alignment of feedback effects from visual cortex to thalamus. *Nat. Neurosci.* 9 1330–1336. 10.1038/nn1768 16980966

[B153] Wang Y. Toledo-Rodriguez M. Gupta A. Wu C. Silberberg G. Luo J. (2004). Anatomical, physiological and molecular properties of Martinotti cells in the somatosensory cortex of the juvenile rat. *J. Physiol. (Lond).* 561 65–90. 10.1113/jphysiol.2004.073353 15331670 PMC1665344

[B154] Weiler N. Wood L. Yu J. Solla S. A. Shepherd G. M. (2008). Top-down laminar organization of the excitatory network in motor cortex. *Nat. Neurosci.* 11 360–366. 10.1038/nn2049 18246064 PMC2748826

[B155] Wertz A. Trenholm S. Yonehara K. Hillier D. Raics Z. Leinweber M. (2015). Single-cell-initiated monosynaptic tracing reveals layer-specific cortical network modules. *Science* 349 70–74. 10.1126/science.aab1687 26138975

[B156] West D. C. Mercer A. Kirchhecker S. Morris O. T. Thomson A. M. (2006). Layer 6 cortico-thalamic pyramidal cells preferentially innervate interneurons and generate facilitating EPSPs. *Cereb. Cortex* 16 200–211. 10.1093/cercor/bhi098 15843627

[B157] Wilson N. R. Runyan C. A. Wang F. L. Sur M. (2012). Division and subtraction by distinct cortical inhibitory networks in vivo. *Nature* 488 343–348. 10.1038/nature11347 22878717 PMC3653570

[B158] Wiser A. K. Callaway E. M. (1996). Contributions of individual layer 6 pyramidal neurons to local circuitry in macaque primary visual cortex. *J. Neurosci.* 16 2724–2739. 8786448 10.1523/JNEUROSCI.16-08-02724.1996 PMC6578755

[B159] Wozny C. Williams S. R. (2011). Specificity of synaptic connectivity between layer 1 inhibitory interneurons and layer 2/3 pyramidal neurons in the rat neocortex. *Cereb. Cortex* 21 1818–1826. 10.1093/cercor/bhq257 21220765 PMC3138515

[B160] Xu X. Callaway E. M. (2009). Laminar specificity of functional input to distinct types of inhibitory cortical neurons. *J. Neurosci.* 29 70–85. 10.1523/jneurosci.4104-08.2009 19129386 PMC2656387

[B161] Yabuta N. H. Callaway E. M. (1998). Functional streams and local connections of layer 4C neurons in primary visual cortex of the macaque monkey. *J. Neurosci.* 18 9489–9499. 9801386 10.1523/JNEUROSCI.18-22-09489.1998 PMC6792868

[B162] Yang W. Carrasquillo Y. Hooks B. M. Nerbonne J. M. Burkhalter A. (2013). Distinct balance of excitation and inhibition in an interareal feedforward and feedback circuit of mouse visual cortex. *J. Neurosci.* 33 17373–17384. 10.1523/jneurosci.2515-13.2013 24174670 PMC3812505

[B163] Yoshimura Y. Callaway E. M. (2005). Fine-scale specificity of cortical networks depends on inhibitory cell type and connectivity. *Nat. Neurosci.* 8 1552–1559. 10.1038/nn1565 16222228

[B164] Yoshimura Y. Dantzker J. L. Callaway E. M. (2005). Excitatory cortical neurons form fine-scale functional networks. *Nature* 433 868–873. 10.1038/nature03252 15729343

[B165] Yoshioka T. Levitt J. B. Lund J. S. (1992). Intrinsic lattice connections of macaque monkey visual cortical area V4. *J. Neurosci.* 12 2785–2802. 1377236 10.1523/JNEUROSCI.12-07-02785.1992 PMC6575826

[B166] Zarrinpar A. Callaway E. M. (2016). Functional local input to layer 5 pyramidal neurons in the rat visual cortex. *Cereb. Cortex* 26 991–1003. 10.1093/cercor/bhu268 25405939 PMC4737602

[B167] Zeki S. Shipp S. (1989). Modular connections between areas V2 and V4 of macaque monkey visual cortex. *Eur. J. Neurosci.* 1 494–506. 10.1111/j.1460-9568.1989.tb00356.x 12106135

[B168] Zhang S. Xu M. Kamigaki T. Hoang Do J. P. Chang W. C. Jenvay S. (2014). Selective attention. Long-range and local circuits for top-down modulation of visual cortex processing. *Science* 345 660–665. 10.1126/science.1254126 25104383 PMC5776147

